# Hyper-Activation of STAT3 Sustains Progression of Non-Papillary Basal-Type Bladder Cancer via FOSL1 Regulome

**DOI:** 10.3390/cancers11091219

**Published:** 2019-08-21

**Authors:** Luisa Benerini Gatta, Laura Melocchi, Mattia Bugatti, Francesco Missale, Silvia Lonardi, Benedetta Zanetti, Luca Cristinelli, Sandra Belotti, Claudio Simeone, Roberto Ronca, Elisabetta Grillo, Sara Licini, Debora Bresciani, Regina Tardanico, Szeman Ruby Chan, Emanuele Giurisato, Stefano Calza, William Vermi

**Affiliations:** 1 Department of Molecular and Translational Medicine, School of Medicine, University of Brescia, 25100 Brescia, Italy; 2 Department of Medical and Surgical Specialties, Radiological Sciences, and Public Health, University of Brescia, 25100 Brescia, Italy; 3 ASST Spedali Civili di Brescia, 25100 Brescia, Italy; 4 Department of Pathology, Fondazione Poliambulanza, 25100 Brescia, Italy; 5 Department of Otorhinolaryngology, Head and Neck Surgery—IRCCS Ospedale Policlinico San Martino, University of Genoa, 16121 Genoa, Italy; 6 Janssen Research and Development, Spring House, Horsham, PA 19044, USA; 7 Department of Biotechnology Chemistry & Pharmacy, University of Siena, 53100 Siena, Italy; 8 Division of Cancer Sciences, School of Medical Sciences, Faculty of Biology, Medicine and Health, University of Manchester, Manchester M13 9PT, UK; 9 Department of Pathology and Immunology, Department of Pathology and Immunology, School of Medicine, Washington University, St. Louis, MO 63130, USA

**Keywords:** STAT3, JAK, basal-type, bladder cancer, MYC, FOSL1

## Abstract

Urothelial bladder cancer (UBC) are classified into luminal and basal subtypes showing distinct molecular features and clinical behaviour. Recent in silico data have proposed the activation on the Signal Transducer and Activator of Transcription 3 (STAT3) as relevant transcription factor in UBC. To answer this question, we have combined the retrospective analysis of clinical samples, functional assays on cell lines, interrogation of public UBC datasets and a murine model of basal-type UBC. Immunohistochemistry on a retrospective UBC cohort uncovered that STAT3 Y705 phosphorylation (pSTAT3) is significantly increased in infiltrating basal-type UBC compared to luminal UBC. In vitro, STAT3 silencing in UBC cell lines significantly reduced tumor cell viability and invasion. Gene expression profile of UBC cell lines combined with the analysis of the Cancer Genome Atlas (TCGA) and GSE32894 UBC datasets showed that increased expression of a set of STAT3 targets predicts basal-type, propensity to local progression and worse prognosis. MYC and FOSL1 represent relevant STAT3 downstream targets, as validated by their co-localization in pSTAT3+ UBC cancer cells. These findings were largely reproduced in the BBN-induced murine model of basal-type UBC. Of note, FOSL1 protein resulted strongly expressed in the non-papillary UBC pathway and FOSL1-regulated transcripts were significantly enriched in the transition from NMIBC to MIBC, as indicated by the interrogation of the GSE32894 dataset. The blockade of the STAT3 pathway might represent a novel treatment option for these neoplasms. Monitoring pSTAT3 and the downstream targets, particularly FOSL1, could provide meaningful levels of UBC stratification.

## 1. Introduction

Bladder cancer (BC) is the fourth most common carcinoma among men in the Western world ranking 13th in terms of mortality rate [1]. BC is largely represented by urothelial carcinoma (UBC) [2]. At presentation, 20–30% of UBC are diagnosed as advanced muscle-invasive forms (MIBCs), whereas the remaining 70–80% are non-muscle invasive (NMIBCs), the latter mainly corresponding to low grade superficial papillary tumors (LGPBCs) [1]. While LGPBCs generally have a good prognosis and an excellent survival rate, the outcome of advanced MIBCs remains very poor. LGPBCs treatment involves conservative transurethral resection followed by intra-vesical chemo- or immunotherapy; however, one of the major challenges of their management is the high propensity to recurrence [3]. In addition, a minor fraction of NMIBCs eventually evolve to MIBC, a switch that cannot be predicted by biomarkers currently used in the clinical practice. Treatment approaches for MIBC are limited to radical cystectomy (with or without neo-adjuvant chemotherapy) and urinary diversion, but only 40% of the patients benefit in terms of survival and the quality of life remains poor [4,5].

UBC can evolve though two divergent carcinogenic pathways including the papillary track (80% of the cases) and the CIS track (20%), exhibiting distinct molecular and morphological features along with different clinical behavior [6]. Molecular studies have proposed the existence of two independent transformation pathways of UBC suggesting distinct cell of origin [7]. In particular, LGPBCs derive from the intermediate cells of the urothelium transformed by a combination of gain-of-function mutation in H-RAS, FGFR3 and PI3KCA. On the other hand, the carcinoma in situ (CIS) pathway arises from basal cells as progenitor transformed by a set of loss-of-function mutations affecting p53, RB and PTEN [7,8]. Based on the transcriptomic profile of the tumor, a new classification of MIBC has been proposed [9,10,11,12]. This classification, similarly to breast cancer [11], stratifies UBC in basal- and luminal-types [13]. These studies have also proposed that UBC types are driven by distinct transcription factors. Among the latter, Signal Transducer and Activator of Transcription 3 (STAT3) has been recently proposed in basal-type UBC by in silico analysis [10], particularly in a subset of MIBCs showing “squamous cell carcinoma-like” features [14].

STAT3 is a latent transcription factor downstream of the Janus Kinase (JAK) molecular pathway and is known to be aberrantly activated in a wide variety of cancers [15]. STAT3 oncogenic activity is crucial in several cellular processes including survival and proliferation, invasion, EMT transition, immune escape and stemness [16]. Several strategies have been developed to target JAK/STAT pathway in cancer, including STAT3 inhibitors and JAK inhibitors [17], showing a heterogeneous degree of success [18].

In the urinary tract, STAT3 activation is relevant in maintaining the basal stem cell compartment of the normal urothelium [19,20]. Various STAT3-dependent pro-tumor functions have been proposed for UBC, including the expansion of cancer stem cells [21]. In addition, chronic interleukin-6 (IL6) stimulation and an autocrine cytokine loop is associated with STAT3 activation in MIBCs [22]. In this study, we expanded the analysis on the clinical and biological relevance of STAT3 activation in UBC. We uncover that STAT3 activation is detected in cancer cells and cells of the microenvironment, particularly in basal-type UBC. STAT3 activation promote basal-type UBC progression by affecting tumor cell viability, proliferation and stromal invasion. These biological activities and their clinical relevance are mediated by a set of STAT3 targets including the transcription factor MYC and FOSL1 and their regulome.

## 2. Results

### 2.1. STAT3 Is Activated in the Early Phases of UBC Invasion

**Figure 1:**
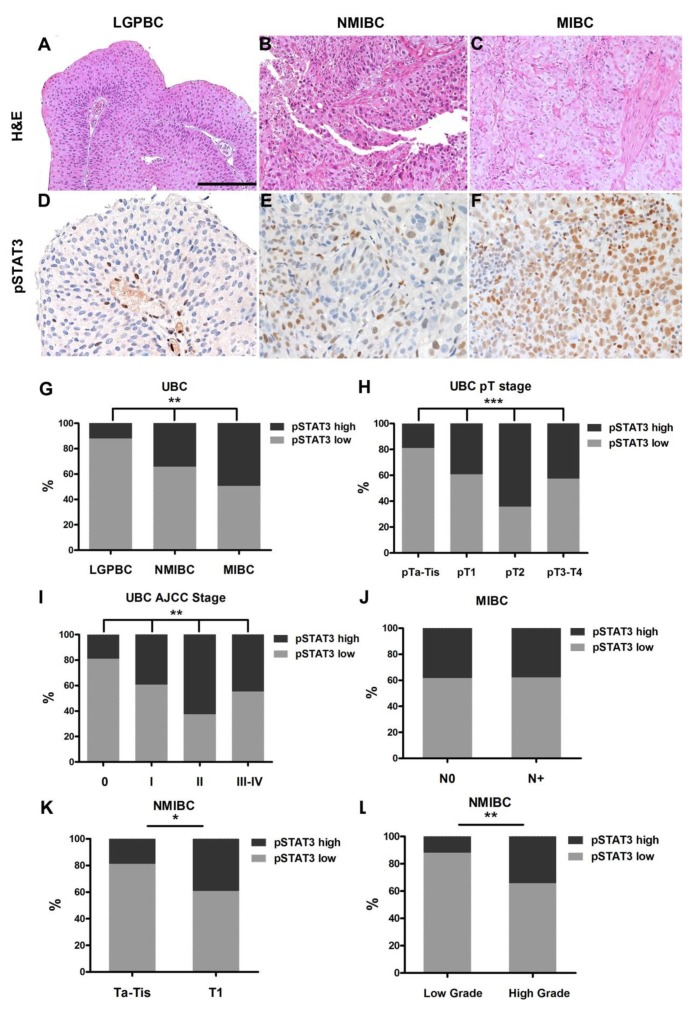
pSTAT3 expression is enriched in MIBCs. Sections are from UBC LGPBC (**A**,**D**), NMIBC (**B**,**E**) and MIBC (**C**,**F**) representative cases and stained as labelled. A significantly increased number of tumor nuclei expressing the phosphorylated forms of STAT3 is observed in MIBCs. Magnification 200×. Scale bar = 100 µm. Sections are counterstained with Meyer’s hematoxylin. In (**G**–**L**), graphs illustrate the percentage of pSTAT3^high^ and pSTAT3^low^ cases in bladder neoplasms sub-grouped for histology type (**G**,**L**), pT (**H**,**K**), stage (**I**), nodal involvement (**J**). Chi-square test was used for all statistical analysis (* *p* < 0.05, ** *p* < 0.01, *** *p* < 0.001).

A cohort of 193 human UBC, including 25 low grade papillary NMIBCs (LGPBCs), 79 high grade NMIBCs and 89 MIBCs, were examined for STAT3 activation by immunohistochemistry (IHC) (Figure 1A–L and Supplementary Tables S1–S4). An antibody recognizing STAT3 phosphorylated at the Y705 residue (pSTAT3) was used. Nuclear expression of pSTAT3 was observed on tumor cells and cells of the microenvironment (Supplementary Figures S1 and S2).

The level of pSTAT3 nuclear expression was measured on tumor cell and evaluated using a four-tired score system as indicated in the Section 4 and illustrated in Supplementary Figure S1. Nuclear expression of pSTAT3 showed a significant increase from NMIBCs to MIBCs. Specifically, a higher fraction of pSTAT3^High^ cases (showing an IHC score 2 or 3) was present in MIBCs compared to NMIBCs and LGPBCs (*p* < 0.01) (Supplementary Table S1, Figure 1G). These findings confirm that STAT3 Y705 phosphorylation is associated with local progression of UBC. We next correlated pSTAT3 expression with clinical and pathological features of the UBC cohort. An increased pSTAT3 level was associated with higher pT (*p* < 0.01) (Supplementary Table S1, Figure 1H) and AJCC stage (*p* < 0.01) (Supplementary Table S1, Figure 1I); on the contrary the gender (*p* = 0.173), age (*p* = 0.458), or node metastasis (*p* = 1) (Figure 1J), did not correlate with pSTAT3 expression (Supplementary Table S1). Interestingly, in NMIBCs pSTAT3 expression increases significantly in the transition from Ta/Tis to T1 tumor (*p* = 0.018) (Supplementary Table S1, 1K), pointing out its potential role as biomarker of early stromal invasion. In addition, pSTAT3 expression is significantly increased in high grade compared to low grade NMIBCs (*p* < 0.01) (Supplementary Table S1, Figure 1L), suggesting a role in the identification of more aggressive transformed cells in the non-muscle invasive setting. The presence of concomitant carcinoma in situ (CIS) was not associated with a significantly different pSTAT3 expression (Supplementary Table S1). 

### 2.2. pSTAT3 Is Selectively Expressed in Basal-Type UBC

**Figure 2:**
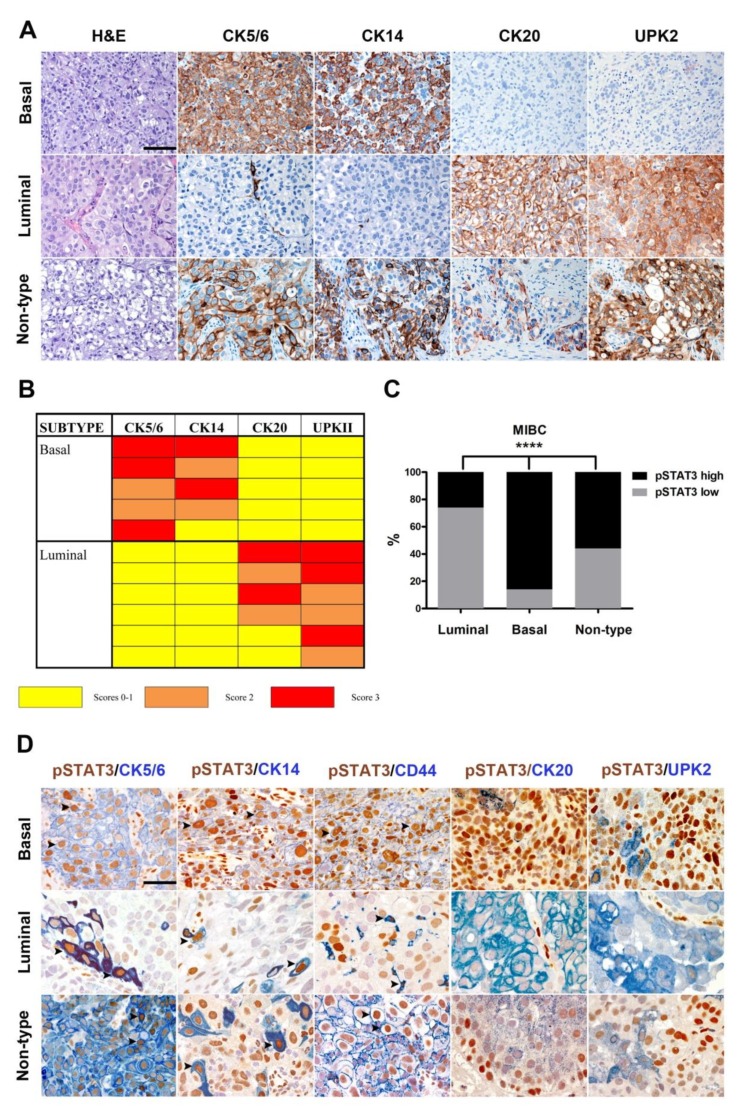
pSTAT3 expression is enriched in basal UBC. Sections are from human MIBCs (**A**,**D**) and stained as labelled. In (**A**), representative basal, luminal and non-type cases are reported as indicated by labels; magnification 200×. Scale bar = 100 µm. In (**B**) a schematic representation of the IHC algorithm used for the classification of luminal and basal cases is illustrated. Basal and luminal cases are called based on the variable combinations of biomarkers as scored using a three-tiered score indicated by color-codes. In (**C**) the graph illustrates the frequency of pSTAT3^high^ pSTAT3^low^ cases in luminal, basal and non-type MIBCs. Chi-square test was used for statistical analysis (**** *p* < 0.0001). In (**D**) double IHC staining illustrate co-expression of pSTAT3 (brown) with basal and luminal markers (blue) in a representative basal, luminal and non-type MIBCs cases. Arrowheads indicate representative double positive cells. Magnification 400×. Scale bar = 50 μm. The latter finding is of interest since STAT3 activation in UBC has been associated with squamous differentiation [14] (Supplementary Tables S2 and S3). Subtype profiling of UBC cell lines was performed using the basal biomarkers CK5/6, CK14, and luminal biomarkers CK20, UPK2. Basal and luminal markers were strongly induced upon heterotopic transplantation in vivo. Indeed, 5637 and HT-1376 xenografts revealed a basal-type profile, T24 cells were non-type, whereas RT4 cells were unique for their luminal profile (Figure 3A). pSTAT3 expression was strong in the transplanted basal cell lines 5637 and HT-1376, but very limited in RT4 luminal cells and T24 cells (Figure 3B).

Based on transcriptomic analysis, STAT3 has been retained as a relevant signal transduction molecule more expressed in a subset of basal-type UBC with squamous differentiation [14]. We tested and verified this hypothesis on a retrospective cohort of MIBCs sub-grouped in luminal-type and basal-type and performed analysis for the expression of pSTAT3. We first classified the UBC cases on TURB (also validated on cystectomy tissue blocks) using a set of validated IHC markers (see Section 4 and Figure 2A,B). Based on this approach, the study cohort was composed of 42 (47%) luminal-type UBC, 21 (25%) basal-type UBC and 26 (28%) “non-type” UBC (Supplementary Table S3). By IHC scoring, basal-type MIBCs showed a significantly higher fraction of pSTAT3^High^ cases compared to luminal and non-type UBC (respectively 86%, 26% and 56%; *p* < 0.0001) (Figure 2C and Supplementary Table S1). By using double IHC, we found that pSTAT3 positive tumor cells co-expressed the basal markers CK5/6, CK14 as well as CD44 (Figure 2D, Supplementary Figure S3). On the contrary, pSTAT3+ tumor cells were regularly negative for the luminal markers UPK2 and CK20 (Figure 2D). It is of note that also STAT3+ cells in the luminal and in the non-type group co-expressed basal markers (Figure 2D). In addition, based on morphology, basal-type UBC with squamous differentiation were strikingly enriched in pSTAT3^High^ cases (*n* = 10/10; 100%; Supplementary Tables S2 and S3). Taken together, these data suggest that in MIBCs, pSTAT3 expression is a strong predictor of basal type.

### 2.3. Persistent Activation of STAT3 Promote Survival, Proliferation and Invasion of Basal-Type UBC Cells

Given that basal-type MIBC frequently display STAT3 activation, we tested the expression of pSTAT3 and surrogate markers of basal and luminal types in four commercially available UBC cell lines, 5637, HT-1376, RT4 and T24, respectively. To this end, IHC was performed on tumor sections obtained from in vivo xenografts (after implantation of UBC cells in immunocompromised mice).

**Figure 3:**
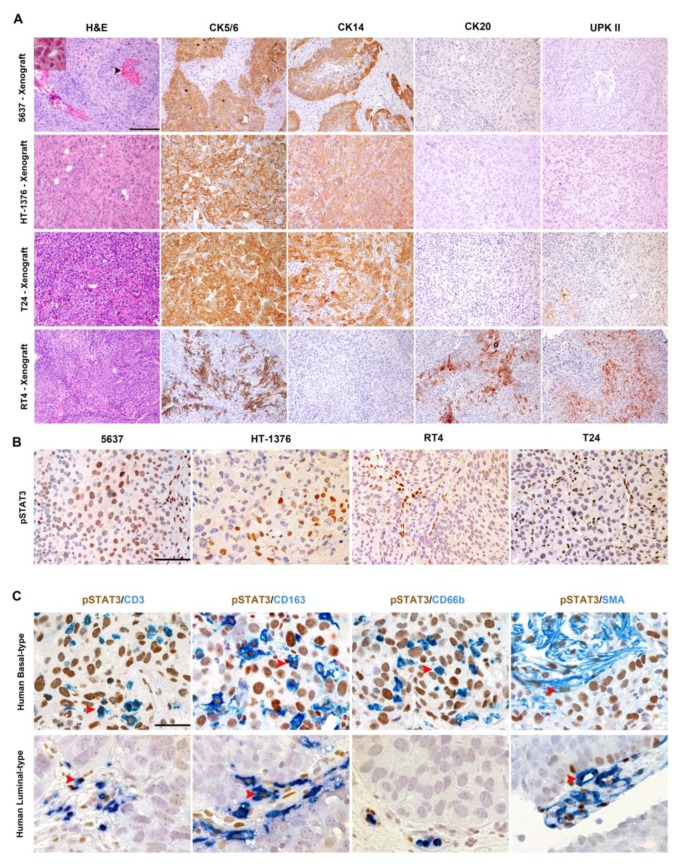
Subtype profiling of UBC cell lines and characterization of pSTAT3+ cells in the microenvironment of UBC. (**A**) Sections are from 5637, HT-1376, T24 and RT4 mouse xenografts and stained as labelled. Magnification of IHC 100×. Scale bar = 200 µm. In (**B**), sections are from xenografts of UBC cell lines stained for phosphorylated STAT3 (pSTAT3). Strong and diffused expression is observed in 5637 and HT-1376 cell lines; magnification 200×. Scale bar = 100 µm. In (**C**), sections are from a basal-type MIBCs and luminal-type MIBCs stained double stained as labelled (pSTAT3/CD3 T-cells, pSTAT3/CD163 macrophages, pSTAT3/CD66b neutrophils and pSTAT3/SMA cancer associated fibroblasts; red arrowheads indicate representative double positive cells. Magnification 400×. Scale bar = 50 µm. High-power view of the inset was obtained at 600× magnification.

All cell lines grow progressively after subcutaneous transplantation showing a morphology consistent with UBC and obvious squamous differentiation (limited to 5637) (Figure 3A and inset).

This suggests that a full basal or luminal phenotype might partially rely on signaling from local cytokines and growth factors. Accordingly, pSTAT3 is regularly expressed in the human tumor microenvironment UBC (Supplementary Figure S2). Particularly, in basal-type UBC, endothelial cells, stromal (SMA+ cancer associated fibroblast) cells and immune cells (including CD163+ macrophages, CD3+ T-cells and CD66b+ neutrophils) react to pSTAT3 (Figure 3C, Supplementary Figure S4), whereas in luminal-type UBC, pSTAT3 was limited to endothelial cells, rare T-cells and some macrophages.

**Figure 4:**
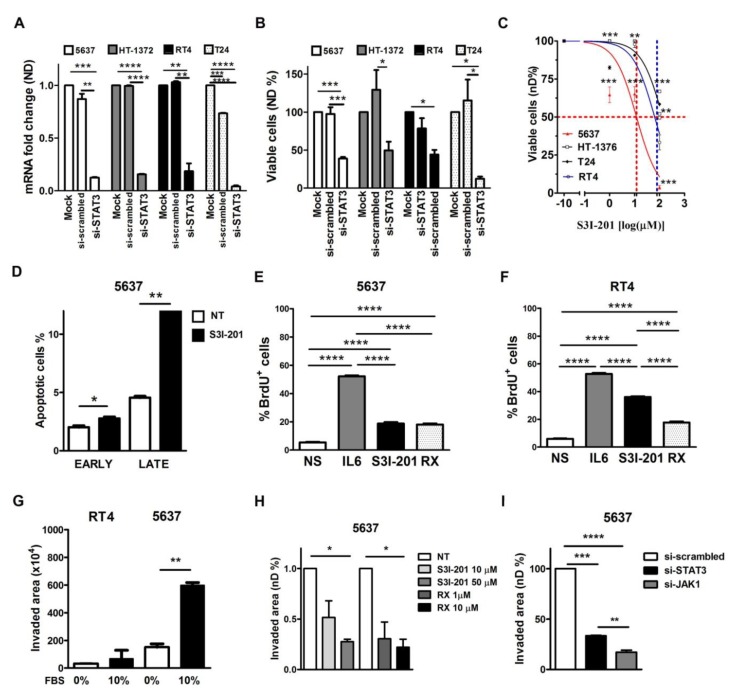
STAT3 blockade in human UBC cell lines. After 144 h of 5 nM STAT3 siRNA delivery, a significant reduction of the STAT3 mRNA (**A**) and of the cell viability (**B**) is observed in all UBC cell lines. A dose-dependent decrease of cell viability is observed after exposure to S3I-201 (1 μM, 10 μM, 100 μM) for 48 h, particularly in 5637 cell line (**C**). The data are expressed as percentage relative to the control. The graph (**D**) shows the cell percentages of apoptotic cells in controls and in S3I-201 treated 5637 cells for 24 h. (**E**,**F**) The graphs shows the effects of STAT3 inhibition of the proliferation in 5637 (**E**) and in RT4 (**F**) UBC cell lines monitored by BrdU incorporation after IL6 stimulation. Cells were pre-treated with 50 μM S3I-201 or 1 μM RX for 2 h and then stimulated with IL6 (100 ng/mL) for 36 h. By matrigel-based invasion assay, the 5637 UBC cell line shows a significantly higher invasive capability (**G**) compared to RT4. S3I-201 (50 μM) or Ruxolitinib (1–10 μM RX) pre-treatment for 24 h (**H**) or genetic ablation of STAT3 or JAK1 (**I**) potently inhibited the basal-type 5637 cell invasion after 24 h. In all graphs the results are the mean ± S.E.M. of three independent experiments. In the graphs (**A**,**B**,**E**,**F**,**H**,**I**) the One-way ANOVA test was used for statistical analysis (* *p* < 0.0.5, ** *p* < 0.01, *** *p* < 0.001, **** *p* < 0.0001). In the graphs C, D and G the Student’s t test was used for statistical analysis (* *p* < 0.0.5, ** *p* < 0.01, *** *p* < 0.001, **** *p* < 0.0001).

The basal-type 5637 cell line showed an increased proliferation rate compared with luminal-type RT4 (Supplementary Figure S5A). To better dissect the role of STAT3, we performed genetic silencing by transient STAT3-specific si-RNA (si-STAT3) delivery. At 5 nM si-STAT3 showed a marked efficiency by reducing the levels of STAT3 mRNA and protein (Figure 4 and Supplementary Figure S5B). A second si-STAT3 (referred as si2-STAT3) was slightly less efficient in knocking-down STAT3 mRNA (Supplementary Figure S5C), limiting its usage in the following experiments. As shown in Figure 4B, knockdown of STAT3 significantly reduced the viability in all UBC cell lines. Moreover, STAT3 knockdown significantly reduced the proliferation of 5637 cells as demonstrated by Crystal Violet Assay and tetrazolium salts-MTS Assay (Supplementary Figure S6). 

The biological role of STAT3 in basal-type 5637 and luminal-type RT4 cell lines was also investigated, using the chemical molecules S3I-201 (NSC 74859) and RX, inhibitors of the JAK/STAT3 pathway. S3I-201 and RX treatment of 5637 and RT4 cell lines for 24 h resulted in impaired phosphorylation of STAT3 (Supplementary Figure S7) by immunoblotting analysis. In addition, a S3I-201 dose-dependent decrease of the cellular viability of all UBC cell lines was observed after 48 h of treatment. This was particularly evident in the basal-type 5637 cell line with an IC50 12.3 µM compared to the luminal RT4 with IC50 of 80 µM (Figure 4C). By using Pacific Blue™ Annexin V/SYTOX™ AADvanced™ assay on S3I-201 treated cells, we could detect an increased percentage of necrotic cells in basal type 5637 vs. luminal type RT4 (Figure 4D and Supplementary Figure S8). In addition, the effect of STAT3 inhibition on tumor cell proliferation was monitored by BrdU incorporation after IL6 stimulation. The basal-type 5637 cells show a significantly reduced response to IL6 stimulation after STAT3 inhibition, greater than luminal-type RT4 cells (Figure 4E,F and Supplementary Figure S9). Many of the STAT3 upstream kinase inhibitors have demonstrated efficacy in cancer, suggesting a therapeutic window for STAT3-dependent cancer. To explore this option in UBC, we used RX, a potent JAK1/JAK2 inhibitor approved for the treatment of intermediate/high risk myelofibrosis [23]. The RX treatment significantly reduces the proliferation rate of UBC cells after IL6 stimulation (Figure 4E,F and Supplementary Figure S9). 

STAT3 activation regulates stromal invasion by modulating genes as TWIST, SNAI1, MMP, FOSL1. Remarkably, increased pSTAT3 expression in clinical samples is associated with stromal invasion and local progression to MIBC (Figure 1 and Supplementary Table S1). To test the role of STAT3 activation in invasion, a cell invasion assay was performed by using Matrigel-coated transwells. Under these experimental conditions, the basal-type 5637 cells showed an increased invasion capability compared to luminal-type RT4 cells (Figure 4G), that was significantly reduced after genetic knockdown (si-STAT3) or pharmacological blockade (S3I-201) of STAT3 (Supplementary Figure S10) as well as by treatment with RX or si-JAK1 (Figure 4H,I). The effects of JAK1 silencing on STAT3 phosphorylation are shown in the Supplementary Figure S11.

### 2.4. STAT3 Signature Predicts Basal-Type UBC with Poor Prognosis

**Figure 5:**
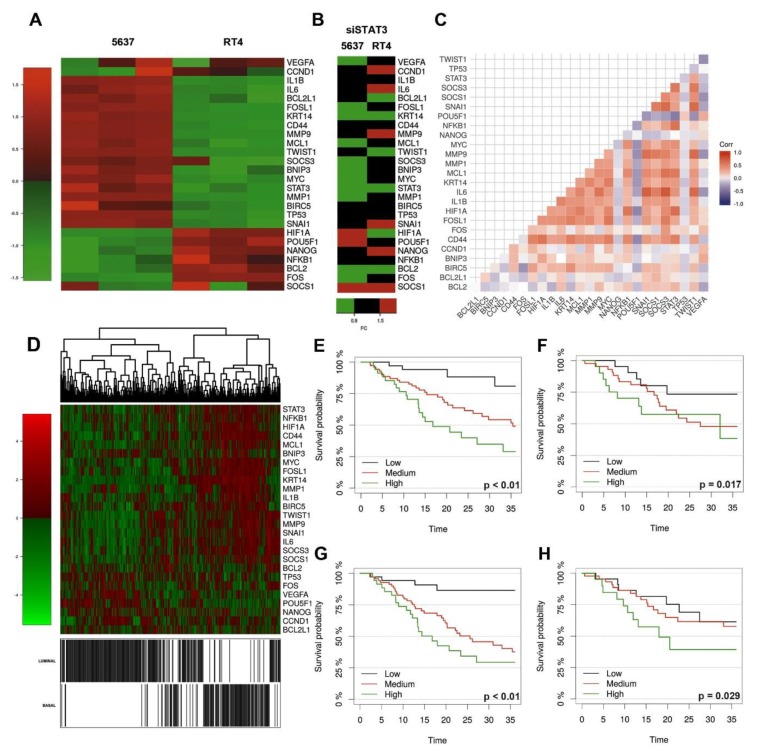
STAT3 drives a unique gene expression profile in basal subtype of UBC. STAT3 signature is significantly enriched in basal-type 5637 UBC cell line (*n* = 3) compared to RT4 cells (*n* = 3) (**A**) and modulated by siRNA STAT3 (**B**). A Student *t*-test was used for statistical analysis of three independent experiments. The heatmap in (**C**) illustrates the pairwise correlation structure among genes in the selected signature. The white background indicates a non-significant (*p* > 0.05) Pearson correlation coefficient. Two main blocks of positive correlation are evident, one correlating FOSL1, HIF1A, IL1B, IL6, KRT14, MCL1, MMP1, MYC among each other and with SNAI1, SOCS1, SOCS3, STAT3. In (**D**), the heat-map with hierarchical clustering of the STAT3 signature illustrates a subtype discrimination (basal vs. luminal) by the signature. Classification was performed using regularized logistic regression via elastic-net algorithm. Kaplan-Meir curves using all genes of the signature with an L2 regularization (**E**–**G**, training set) and a genes subset selected via bootstrap (**F**–**H**, test set); survival curves are derived by clustering samples around lower, median and higher values (labelled as low, medium, and high) of the continuous predictor using a nearest-neighbor estimate.

pSTAT3 modulates a large set of genes involved in biological processes affecting fully transformed cancer cells and their precursors [15]. Using a Pubmed search we identified a list of largely validated STAT3 targets (Supplementary Table S5). The STAT3 gene signature was tested in the basal-type 5637 cell line and the luminal-type RT4 cell line. Using cell starvation with low serum (1%), the basal-type 5637 cell line showed an increased level of numerous STAT3 targets compared to luminal-type RT4 cell line (Figure 5A and Supplementary Table S6). Among STAT3 targets, IL6, IL1B, MYC, FOSL1, TP53, TWIST, SNAI1, BCL2L1, MCL1, BNIP3, BIRC5, CD44, KRT14, MMP1 and MMP9 were significantly over-expressed in the basal-type 5637 compared to the luminal-type RT4; conversely HIF1A and FOS were over-expressed in RT4 cells.

To further define the list of STAT3 modulated genes in the UBC setting, the STAT3 gene signature was also obtained after genetic silencing using si-STAT3. Compared with si-RNA-scrambled, si-STAT3 significantly modulated FOS (FC = 0.57, FDR = 0.02), FOSL1 (FC = 0.60, FDR = 0.041), MCL1 (FC = 0.47, FDR = 0.001) and KRT14 (FC = 0.39, FDR = 0.02) in basal-type 5637 cells; MYC and MMP1 were also affected, but did not pass the FDR ≤ 0.05 threshold. On the contrary, only the HIF1A gene was significantly modulated by STAT3 (FC = 0.11, FDR = 0.0006) in the luminal-type RT4 cells (Figure 5B and Supplementary Table S6). A more limited fraction of STAT3 modulated genes was affected by si-STAT3 in the basal-type HT-1376 cells and in the non-type T24 cells (Supplementary Table S6). These findings indicate variability within basal-type UBC in modulating STAT3-dependent targets. We thus expanded our analysis in silico and tested the clinical relevance of the STAT3 gene signature in public available dataset on the transcriptome analysis of large UBC cohorts. To this end we used the TCGA RNA-seq dataset containing 388 UBC samples [9]. Targets within our STAT3 signature showed a significant level of correlation (Figure 5C). In addition, clustering analysis showed that genes belonging to STAT3 signature were significantly over-expressed in basal-type UBC (Figure 5D) as previously shown by others using a more expanded gene list [10].

Using a regularized logistic regression model on a subset of the samples (training set), we identified BIRC5, CD44, FOS, FOSL1, HIF1A, KRT14, MMP9, MYC, POU5F1, SNAI1, SOCS1, SOCS3, STAT3 as the best predictors of the basal-type UBC with a test set accuracy of 0.94 (CI 95% 0.88–0.98), sensitivity of 0.88 and specificity of 0.97 (Supplementary Table S7, Supplementary Figure S12). Among these, BIRC5, CD44, KRT14, MYC, SOCS1 and STAT3 were the more stable in the Lasso based variable selection, and a model restricted to these features achieved a high accuracy (0.91; CI 95% 0.85–0.96) on the validation set. Applying the same procedure to an external independent dataset (GSE32894) [12], we confirmed the predictive power for “basal-type” of the signature achieving a 0.93 accuracy (CI 95% 0.89–0.96), with 0.84 sensitivity and a 0.95 specificity.

We thus tested the clinical relevance of the STAT3 signature in the TCGA dataset. Using univariate Cox models, we identified the following genes belonging to STAT3 signature, to be significantly associated to a worse overall: FOS, FOSL1, KRT14, MMP9, MYC, POUF5F1, SNAI1, SOCS3, STAT3, TWIST1 and marginally VEGFA (Supplementary Table S8). To evaluate prediction performance of the STAT3 signature in terms of overall survival we fitted regularized Cox models with Ridge penalization both on the whole signature and on a subset of genes selected via bootstrap (FOS, FOSL1, IL1B, MMP1, NANOG, POU5F1, SOCS1, STAT3, VEGFA).

Cross-validated Brier score over a three-year period evaluated on training set showed a slightly better performance of the gene signature compared to a null model (no genomic information) (Integrated Brier Score, IBS, 0.164 vs. 0.180, 8.5% reduction, Supplementary Figure S13A). This was confirmed on the test set (IBS 0.170 vs. 0.178, Supplementary Figure S13B). To illustrate the distribution of OS given by the different prediction models used, conditional Kaplan-Meier estimates were generated both for the training and test set.

For illustration purposes, estimates are shown for low, medium, and high levels of predictors (Figure 5E–H). We identified a nearly statistically significant (adjusted *p* = 0.09) cut point at approximately the 40th quantile of the distribution of the full signature (Ridge), separating patients with good or bad prognosis, and similarly for the subset signature (Boot Lasso), with a cut point at approximately the 60% quantile of the distribution of the signature (Supplementary Figure S14A,B).

In summary, our analysis suggests that STAT3 signature is enriched in basal type UBC and can predict worse prognosis, as previously proposed [24].

### 2.5. Expression of MYC and FOSL1 Proteins Is Enriched in Basal-Type UBC

**Figure 6:**
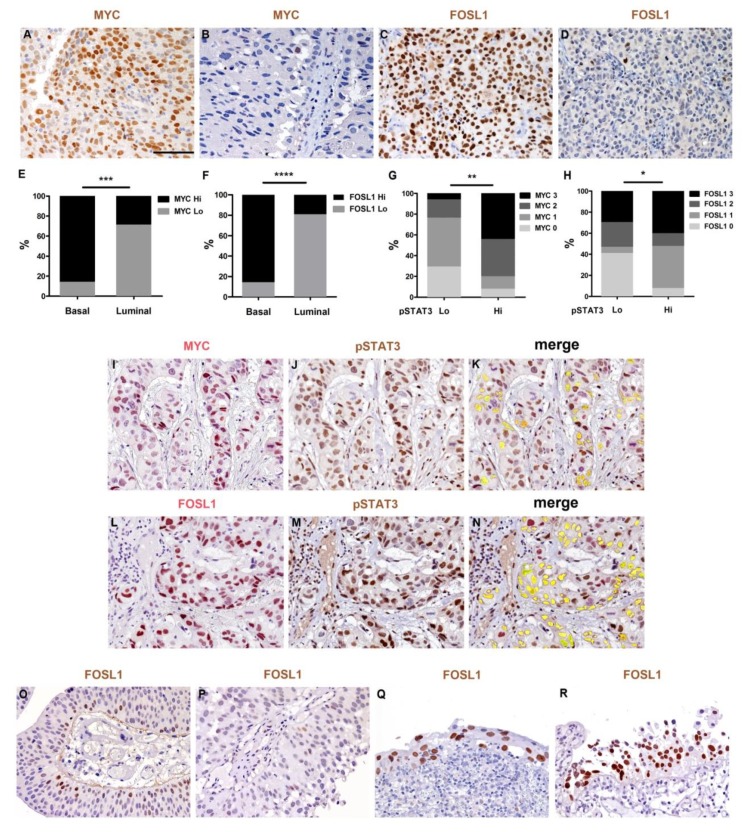
MYC and FOSL1 expression is enriched in basal MIBCs. Sections are from human MIBCs of basal (**A**,**C**) and luminal (**B**,**D**) type and stained as labelled. A significantly increased number of tumor nuclei expressing MYC and FOSL1 is observed in basal type MIBCs; magnification 200×. Scale bar = 100 µm. Graphs illustrate the percentage of MYC high and low MIBCs (**E**) and FOSL1 high and low MIBCs (**F**) sub-grouped for molecular subtype. In G, H the graphs illustrate the percentage of pSTAT3 high and low cases in UBC sub-grouped for MYC (**G**) and FOSL1 (**H**) score. (**I**–**N**) Sections are from basal-type MIBCs. Panels illustrate the sequential double staining of pSTAT3 (brown) with MYC (red in **I**,**J**) or FOSL1 (red in **K**,**L**), as labeled. Images are snap-shot from digital slides at 200X magnification. Cells strongly co-expressing pSTAT3 with MYC or FOSL1 are highlighted in yellow in (**K**,**N**) respectively. (**M**,**N**) Sections from human papillary low grade NMIBC and flat urothelial carcinoma in situ (**O**,**P**) stained for FOSL1. While FOSL1 reactivity is negligible in papillary low grade NMIBC and restricted to basal cells of the papillae (**M**,**N**) numerous FOSL1 positive transformed cells are observed in carcinoma in situ (**O**,**P**); magnification 200×. Chi-square test was used for statistical analysis (* *p* < 0.05, ** *p* < 0.01, *** *p* < 0.001, **** *p* < 0.0001).

To confirm and further extend our in vitro and in silico data, we investigated the expression of selected STAT3-targets on clinical samples. To this end, we performed IHC for the detection of MYC and FOSL1 proteins on 42 MIBCs including 21 basal-type and 21 luminal-type cases. At variance with pSTAT3, MYC and FOSL1 expression is mainly limited to the nuclei of transformed cell. Nuclear MYC and FOSL1 positivity in tumor cells was evaluated using a four-tiered scoring system (Section 4) and cases were classified as low (score 0–1) and high (score 2–3). We found that basal-type MIBCs expressed significantly higher levels of MYC (85.7% vs. 28.6% MYC^high^, *p* = 0.0003) and FOSL1 (85.7% vs. 19.1% FOSL1^high^, *p* < 0.0001) compared to luminal-type cases (Figure 6A–F). Moreover, MYC and FOSL1 expression showed a strong correlation with pSTAT3 levels (respectively *p* = 0.003 and *p* = 0.01) (Figure 6G,H) in basal type UBC. Using double sequential immunostaining, we could confirm co-expression of these two markers on a significant fraction of pSTAT3 expressing tumor cells (Figure 6I–N). Finally, by western blotting we confirmed that S3I-201 and si-STAT3 treatment of 5637 resulted in reduced MYC and FOSL1 protein levels in vitro (Supplementary Figure S15).

**Figure 7:**
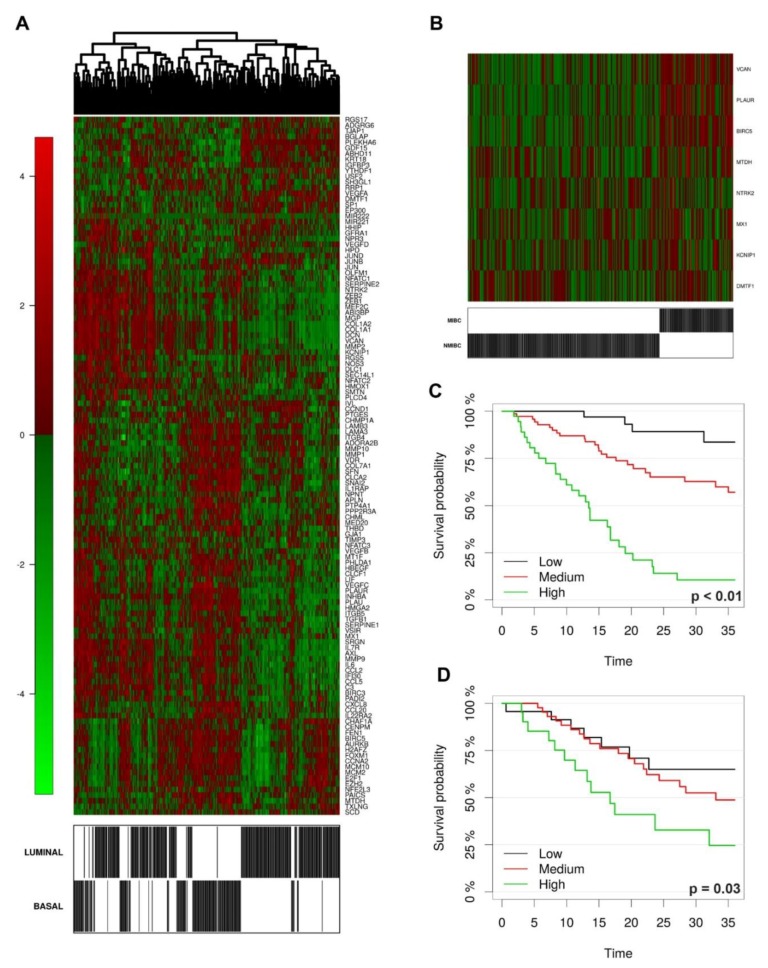
FOSL1 signature is enriched in basal-type UBC and predict poor prognosis. (**A**) Heatmap with hierarchical clustering showing the FOSL1 signature in basal and luminal UBC (TCGA dataset). (**B**) Heatmap illustrating a set significantly overexpressed FOSL1 targets in MIBC vs. NMIBC samples (GSE32894 dataset). (**C**,**D**) Kaplan-Meir curves for FOSL1 signature (“Boot Lasso”) on training (**C**) and test set (**D**) in the TCGA dataset.

Recent data suggest a relevant role of FOSL1 in cancer motility, invasion and EMT transition in several types of human cancer [25,26,27]. Accordingly, FOSL1 regularly resulted negative in low grade papillary NMIBC (FOSL1^high^ = 0/20; 0%), although flat carcinoma in situ showed strong and heterogeneous expression in a fraction of cases (*n* = 8/11; Figure 6M–P). We expanded this observation by testing the expression of a FOSL1 gene signature both in the TCGA and GSE32894 datasets. As expected, FOSL1 signature is enriched in basal-type UBC in both datasets (Figure 7A and Supplementary Table S9), and significantly over-expressed in MIBC compared to NMIBC (GSE32894 dataset), with the lowest level of expression detected in the pTa subgroup (Supplementary Table S10). Using a model based on regularized logistic regression in the GSE32894 dataset, we evaluated the predictive power of the whole FOSL1 signature in classifying MIBC versus NMIBC samples. Due to the relatively small sample size we didn’t split the data in training and test sets, and evaluated prediction performance based on bootstrap estimate, achieving an accuracy of 78.2% (bootstrap CI95% 71.3–84.4%). Within the FOSL1 signature, the most stable transcripts were represented by KCNIPI, NTRK2, BIRC5, MTDH, DMTF1, PLAUR, VCAN, MX1 (Figure 7B and Supplementary Table S11). When considering overall survival, FOSL1 signature showed a potential predictive role for prognosis, as confirmed by a reduction, though slight, in cross validate Brier Score (15.3% vs. 18%, 15% reduction) (Supplementary Figure S16A,B).

The result is confirmed in the test set (Figure 7C,D). Similarly to STAT3 signature, we identified a marginally statistically significant cut point at approximately the 90th quantile of the distribution of the signature, separating patients with good or bad prognosis (Supplementary Figure S17).

**Figure 8:**
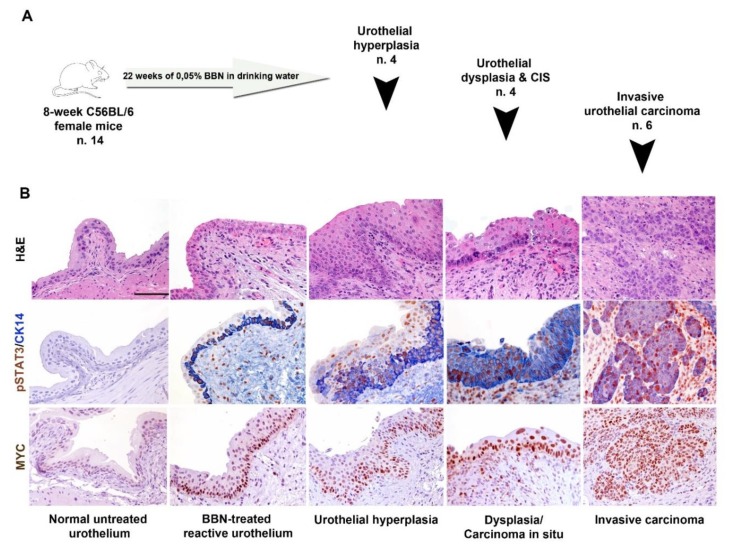
Histology and expression of STAT3, MYC and CK14 on the BBN-murine model. Section are from mice treated for 22 weeks with 0.05% BBN administration in drinking water as illustrated in A and stained as labelled. Magnification 200×. Scale bar = 100 µm.

N-butyl-N-(4-hydroxybutyl)-nitrosamine (BBN) is the most widely used urothelial chemical carcinogen. The spectrum of urothelial lesions induced by BBN is variable, but it largely mimics the histology of basal subtype observed in humans [7,28,29]. Thus, we investigated the expression of pSTAT3, basal (CK14, CK5/6) and luminal (CK20 and UPK2) markers as well as MYC by IHC in the BBN-induced mouse UBC model (Figure 8). CK20 and UPK2 are expressed by the umbrella cells lining the normal bladder lumen; only basal cells show positivity for the basal marker CK5/6 (data not shown), whereas pSTAT3, CK14 and MYC resulted regularly negative (Figure 8).

After 22 weeks of BBN exposure, normal urothelial start acquiring positivity for CK5/6 (data not shown). Upon cell transformation, a progressive increase in the number of pSTAT3+ CK14+ MYC+ cells was observed from precursors to invasive carcinoma (Figure 8). On the contrary, transformed cells showed no reactivity for the luminal markers CK20 and UPK2 (not shown). These findings confirm that basal-type UBC progress to fully invasive cancer through the expansion of pSTAT3+ basal-type cells.

## 3. Discussion

Recent genomic studies have shed new light on the heterogeneity of urothelial bladder cancer (UBC) [9,10,11,12]. Two major molecular subtypes, namely basal and luminal UBC, have been consistently identified in terms of genomic abnormalities, pathway activation, immune-contexture and clinical behaviour. Here, we report the characterization of the role and clinical significance of Signal Transducer and Activator of Transcription 3 (STAT3) in basal UBC. We uncover that STAT3 activation, as measured on clinical samples using an antibody recognizing STAT3 phosphorylated at the Y705 residue (pSTAT3), is expressed in tumor cells and cells of the microenvironment, occurs in the very early phases of mucosal infiltration and increases over local progression to advanced MIBC. In addition, the frequency of pSTAT3+ cancer cells is significantly higher in the basal-type UBC compared to luminal-type. By modulating the level of STAT3 expression on human UBC cell lines, we could demonstrate a relevant role for STAT3 in tumor cell viability, proliferation and invasion. These biological activities and their clinical relevance in predicting worse prognosis are mediated by a set of STAT3 targets, as identified by using an “in silico” approach on publicly available UBC datasets. Among STAT3 targets, we could validate MYC and FOSL1 at the protein level, on either human or murine basal-type UBC. Based on a set of additional computational results, we propose that the STAT3-dependent expression of FOSL1 and its downstream targets marks the very early phases of UBC progression by modulating cell migration and invasion.

STAT3 is aberrantly activated in a wide variety of cancers [15] and regulates several biological processes during cell transformation and cancer progression. STAT3 activation promotes stromal invasion and cell migration thus favouring local and distant spread [30]. The first major finding of this study is the identification of the role of pSTAT3 in local progression of UBC. Accordingly, the frequency of pSTAT3+ tumor cells is significantly higher in MIBC compared to NMIBC and positively correlates with pT stage, showing the lowest level in pTa/Tis subgroups. Furthermore, by using a Matrigel-coated transwell system as in vitro assay, we could demonstrate a STAT3-dependent (by siRNA and chemical inhibition) invasion capability of the 5637 basal-type cells line compared to RT4 luminal-type cells lines. Finally, among STAT3 targets, the 5637 basal-type cell line showed a significant up-regulation of TWIST, SNAI1, MMP1 and MMP9, and FOSL1, all well-known regulators of cell migration and invasion [27]. MMP1 and FOSL1 were also significantly down-regulated by STAT3 genetic silencing in the 5637 basal-type line. Of note, in clinical practice, no reliable prognostic markers can predict stromal and muscle invasion of UBC. Recent advances in NMIBC taxonomy have proposed three main subgroups showing basal- and luminal-like features with different clinical outcomes [31]. We envisage that surrogate biomarkers for STAT3 activation (including pSTAT3 or selected STAT3 targets) might offer an additional tool for better prognostication and patient selection to appropriate treatment schedules.

The second relevant observation of this study is the restricted expression of pSTAT3 in basal-type UBC compared to the luminal-type counterpart. pSTAT3 activation has been already documented in basal-type UBC by using transcriptomic analysis [10,14,32]. Here we resolved this issue at the single cell level by using immunohistochemistry. We found that enrichment for STAT3 targets in basal type UBC, as measured by transcriptome analysis is paralleled by a significantly increased fraction of cancer cells expressing pSTAT3+ in basal-type UBC. The tumour initiating cells in UBC display a basal phenotype [19,28,33]. In the basal-type [29] BBN-induced UBC murine model, we found that STAT3 phosphorylation occurs early in UBC precursors and is accompanied by co-expression of basal cytokeratin and nuclear MYC. It should be reminded that STAT3 regulates the expression of many basal cytokeratins [19]. In human UBC samples, the expression of basal and luminal markers is heterogeneous ([32] and this study) and a set of luminal-type UBC contains a minor fraction of pSTAT3+ infiltrating tumor cells co-expressing basal markers.

These findings might suggest that STAT3+ tumor-initiating cells might also continuously fuel the differentiated compartment of a fraction of luminal-type UBC.

In this scenario, the dominant phenotype of each UBC case likely depends on the co-occurring genomic background of cancer cells or on the microenvironment contexture. Within the group of basal-type UBC, those with squamous differentiation, as defined by morphology, display an aggressive phenotype and show the highest level and frequency of STAT3 activation [14]. In keeping, the human 5637 cell line undergoes obvious squamous differentiation coupled with high STAT3 activation upon in vivo transplantation; in addition, UBC cases with squamous differentiation in our retrospective cohort invariably resulted STAT3^high^. Based on this finding, we propose that, in most of these cases, STAT3 activation cooperates with other pathways to commit basal cell progenitor toward squamous cell differentiation [34]. As measured by the gene expression profile, a large fraction of STAT3 targets is up-regulated in the basal-type 5637 line compared to the luminal-type RT4. Further, by genetic silencing of STAT3, we could significantly down-regulate FOSL1, FOS, MCL1 and KRT14. To define clinically relevant STAT3 targets, we interrogate the TCGA and the GSE32894 UBC datasets [9,12]. This approach confirmed the identification of a set of STAT3 targets highly predictive of basal UBC. These include STAT3, BIRC5, CD44, SOCS1, MYC, KRT14 among others. In addition, the analysis performed on the TCGA dataset showed that the expression of STAT3 targets has some potential prognostic value, thus suggesting new biomarkers of clinical relevance for the identification of aggressive UBC cases. Previous analysis, also using a different STAT3 signature, proposed a similar finding [12].

Among relevant STAT3 targets, we validated the expression of MYC and FOSL1 at the protein level. In our retrospective cohort, a significantly higher fraction of MYC+ and FOSL1+ tumor cells were detected in basal-type UBC compared to luminal-type (Figure 6). Moreover, the expression level of both targets correlates with the level of pSTAT3. Accordingly, by using double sequential immunostaining, a significant fraction of STAT3+ tumor cells in a set of basal-type MIBC co-expressed MYC and FOSL1. The pro-tumorigenic role of MYC in UBC has been already proposed [35]. While the MYC gene is amplified in a small fraction of aggressive UBC, its over expression at the protein level is recurrent [36], particularly in the basal group (this study), thus suggesting additional mechanisms of over-expression. 

STAT3-inducible elements have been identified in the promoter region of human FOSL1 gene. Moreover, chip-PCR analysis showed that STAT3 bind directly the FOSL1 gene promoter in cancer cell lines stimulated with IL6 [26]. Recent data suggest a relevant role of FOSL1 in cancer motility, invasion and EMT transition in several types of human cancer [14]. FOSL1 promotes tumor invasion of triple negative breast cancer [37] and is efficiently inhibited by the multiple kinase inhibitor SKLB646 [38]. The role of FOSL1 in UBC is still largely unexplored. It has been proposed that FOSL1 control the motility of bladder cancer cells via transcriptional up-regulation of the receptor tyrosine kinase AXL [27], which is also involved in squamous cell carcinoma growth through c-Jun activation [39]. FOSL1 is also regulated by miRNA-34, a microRNA with a key role in cancer stemness, metastasis and chemo-resistance [39]. In our cohort, FOSL1 protein expression regularly resulted negative in Ta/Tis NMIBCs. Accordingly, FOSL1 targets are significantly up-regulated following stromal and muscle invasion in UBC clinical samples, as testified by the interrogation of the GSE32894 dataset. Among the latter, KCNIPI, NTRK2, MTDH, DMTF1, PLAUR, VCAN have been clearly implicated in cancer invasion and metastasis. Of note, FOSL1+ cells were recurrently detected in flat carcinoma in situ, suggesting that its role could be restricted to CIS-MIBC track. Finally, FOSL1 expression and its target predict poor overall survival. All these findings point toward a role of FOSL1 as major STAT3-dependent regulator of stromal and muscle invasion in basal UBC. 

In summary, data from this study propose that basal-type UBC and their precursors represent candidates to STAT3 blockade. In the UBC microenvironment, pSTAT3 is also expressed by endothelial cells as well as cancer associated fibroblasts, macrophages, neutrophils and T-cells. On the contrary, MYC and FOSL1 are restricted to cancer cells. This differential pattern of expression suggests that in UBC STAT3 activation might rely on genomic and epigenetic abnormalities (limited to cancer cells) as well as on ligand dependent activation of cytokine and growth factor receptors (on cancer cells and cells of the microenvironment). Among the latter, IL6R, EGFR and FGFR should be considered, with some of them already targeted [40]. The blockade of the STAT3 pathway at a different level might thus benefit from combined approaches, approved or under investigation [18], targeting cancer cells and the cell of the microenvironment. It should be restated that resistance to STAT3 blockade in many cancer types can be acquired by MEK activation, suggesting combined STAT3/MEK inhibition. UBC are also responsive to check point inhibitors [41]. However, only a limited fraction of responder can be identified up-front. Basal-type UBC with squamous differentiation contain dense T-cell infiltration and express CD274 (PD-L1), thus suggesting susceptibility to PD1/PD-L1 inhibition; however, occurrence of an EMT signature on stromal cells has shown a detrimental effect in this subgroup [42]. Notably, STAT3 (and FOSL1) blockade might relieve immunosuppression and reverse EMT providing a strong rational for combination therapy with immune checkpoint inhibitors in the STAT3+ basal UBC.

## 4. Material and Methods

### 4.1. Patient Tissues and Controls

Formalin fixed paraffin-embedded (FFPE) UBC and control biopsies were retrieved from the archive of the Department of Pathology, ASST-Spedali Civili, Brescia, Italy. This retrospective study was conducted in compliance with the Helsinki Declaration and with policies approved by the Ethics Board of ASST Spedali Civili di Brescia (IRB code: NP 2483/2016 to WV). Biopsies from 2000 to 2016 were reviewed and classified according to the World Health Organization (WHO) histologic grading system [43] and staged according to the TNM staging system [44]. The cohorts consisted of diagnostic biopsies from 104 NMIBCs (stage Ta, Tis, T1), and 89 MIBCs (stage ≥ T2); 140 patients (51 NMIBCs and 89 MIBCs) underwent cystectomy. Clinical follow-up data were retrieved from the Department of Urology ASST Spedali Civili di Brescia. Based on the clinical behaviour, high grade NMIBCs were also divided into two groups including a group with stable disease (Ta, Tis, T1; *n* = 53) and the second group with histological progression to muscle-invasive disease at clinical follow up (≥T2; *n* = 26). The control group consisted of five patients presenting with bladder dysfunctional diseases and showing histologically normal or reactive urothelium (NU). pSTAT3 expression was tested in biopsies obtained by endoscopic transurethral resection (TURBT) immediately formalin-fixed for 14 h. Classification of MIBCs into luminal and basal subtypes was performed as described below in details. Exclusion criteria included any treatment before diagnostic biopsy and occurrence of other malignancies. Demographic, histological, and clinical findings are summarized in Supplementary Tables S1–S4.

### 4.2. Immunohistochemistry (IHC) and Immunocytochemistry (ICC)

Four-micron thick FFPE sections were obtained from human tissue biopsies (retrospective cohort) and tissue blocks obtained from xenograft of human cell lines and BBN murine model. Heat mediated antigen retrieval was performed in a microwave oven and endogenous peroxidase activity was quenched using 0,3% hydrogen peroxide (Sigma-Aldrich, Saint-Louis, MO, USA) diluted with methanol (Sigma-Aldrich). After washing with Tris-Buffered Saline (TBS, Sigma-Aldrich) solution, slides were incubated with the primary antibody for 1 h at room temperature and revealed by a 30 min incubation with a horseradish-peroxidase polymer (Envision+ Dual Link System, Agilent Technologies, Santa Clara, CA, USA, or Novolink Polymer Detection System (Leica Biosystems, Wetzlar, Germany), followed by 3,3′-diaminobenzidine (Leica) as chromogen. Sections were counterstained with Mayer’s haematoxylin (Bioptica). For double staining, after completing the first immune reaction, the second reaction was visualized using Mach 4 MR-AP (Biocare Medical, Concord, CA, USA), followed by Ferangi Blue (Biocare) as chromogen. Primary antibodies are reported in Supplementary Table S12. pSTAT3, CK5/6, CK14, CK20, MYC and FOSL1 staining were evaluated by a semi-quantitative method using the following score: score 0 ≤ 5% of positive tumor cells: score 1 = 5–25% positive tumor cells; score 2 = 25–50% positive tumor cells; and score 3 ≥ 50% of positive tumor cells (Supplementary Figure S1). Nuclear reactivity on endothelial cells represented an internal positive control for pSTAT3 expression [45]. For MYC and FOSL1 stain with an isotype control and omission of the primary antibody was performed (Supplementary Figure S18). For UPK2 a lower cut-off of positivity was used as follow: score 0 = negative; score 1 ≤ 10% of positive tumor cells; score 2 = 10–25% positive tumor cells; score 3 ≥ 25% of positive tumor cells, as this is a highly specific marker of terminal urothelial differentiation [46].

Double sequential immunostains were performed on three cases of basal-type MIBCs. Briefly, the first reaction is delated after first chromogen de-stain and stripping. The antibodies for the first immune reaction (MYC and FOSL1) were revealed using Novolink Polymer and developed in 3-amino-9-ethylcarbazole chromogen (AEC), counterstained with haematoxylin and cover-slipped using gelatin. The slides were then digitally scanned using Aperio Scanscope CS (Leica Mycrosystems). After cover slip removal AEC was washed out and the slides were eluted using a 2-Mercaptoethanol/SDS solution (20 mL 10% *w*/*v* SDS with 12.5 mL 0.5 M Tris-HCL, pH6.8, 67.5 mL ultra-pure water and 0.8 mL 2-ME). Slides were subsequently incubated in this solution in a shaking water-bath pre-heated at 56 °C for 30 min. Sections were washed for 1 h in distilled water. After unmasking in microwave, pSTAT3 is revealed in 3,3′-diaminobenzidine, counterstained with haematoxylin, cover-slipped and digitally scanned. The two digital slides were processed using ImageScope. Slides were synchronized and corresponding tumor areas were taken using the snapshot and processed using the counter tool.

### 4.3. Assignment to UBC Subtypes

The classification of MIBCs into subtypes was performed on UBC TURB by IHC using a four marker panel composed of CK5/6 (basal marker), CK14 (basal marker), CK20 (luminal marker) and UPK2 (luminal marker), as indicated by gene expression profiling [9,10,11,12] and IHC [14,32,47]. We developed an algorithm based on predominant marker expression (Figure 2B). According to this, we classified MIBCs as “basal-type” when expressing (i) high CK5/6 or CK14 (score 3) or (ii) moderate CK5/6 and CK14 expression (score 2) in the absence/low level of luminal markers (score 0–1). We defined MIBCs as “luminal” when expressing i) high CK20 (score 3) or UPK2 (score 2–3), or (ii) moderate/low CK20 and UPK2 (score 1–2), with only absent/focal basal markers (score 0–1). Cases outside the above-mentioned criteria were classified as “non-type”. Subtype human MIBCs cell lines (HT-1376, 5637, T24 and RT4) were defined using the same IHC panel. IHC staining was evaluated by estimating the percentage of positive tumor cells.

### 4.4. BBN-Mouse Model and Xenograft Models of Human UBC Cell Lines

The organ-specific carcinogen N-butyl-N-(4-hydroxybutyl)-nitrosamine (BBN, cat. No. B8061, Sigma Aldrich) has been given in drinking water (0.05%) for 22 weeks to eight-week-old wild-type (C57BL/6) female mice (*n* = 14). At the end of the experimental procedure, bladder have been explanted, formalin fixed and paraffin-embedded for histology evaluation. BBN-induced pathology was classified as previously reported in hyperplasia, carcinoma in situ and infiltrating carcinoma [29]. IHC staining for pSTAT3, CK5/6, CK20, CK14, UPK2 and MYC were performed as indicated above. For xenograft models of human UBC cell lines, six-week-old NOD/SCID mice (Envigo, Udine, Italy) were inoculated s.c. into the dorsolateral flank with 5 × 10^6^ tumor cells in 200 µL of PBS. When reached the volume of 500 mm^3^ tumors were excised, formalin fixed, and paraffin embedded for histology.

Animal experiments were approved by the local animal ethics committee and were performed in accordance with national guidelines and regulations. Procedures involving animals and their care conformed with institutional guidelines that comply with national and international laws and policies (EEC Council Directive 86/609, OJ L 358, 12 December 1987) and with “ARRIVE” guidelines (Animals in Research Reporting In Vivo Experiments).

### 4.5. Cell Cultures

T24 (ATCC^®^ HTB4TM), RT4 (ATCC^®^ HTB2TM), HT-1376 (ATCC^®^ CRL1472 TM), 5637 (ATCC^®^ HTB9TM) cell lines were obtained from ATCC-LGC Standards Repository (Rockville, USA). T24 and RT4 cells were maintained in ATCC-formulated McCoy’s 5a Medium Modified (cat. no. 26600-023, GibcoTM for Life Technologies—Thermo Fisher Scientific, Waltham, MA, USA). HT-1376 cells were cultured in ATCC-formulated Eagle’s Minimum Essential Medium (cat. No. 11095-080, GibcoTM). 5637 cells were maintained in ATCC-formulated RPMI1640 Medium (cat. No. A10491-01, GibcoTM). All media were supplemented with 10% fetal bovine serum (FBS) (cat. no. S0115, Biochrom, Berlin, Germany), 1% Penicillin/Streptomycin (cat. No. 15070-063, GibcoTM), and the cells were cultured at 37 °C and 5% CO_2_. The cell lines were thawed and at least three passages were performed before their use; cells were passed at 80% confluency. The cell lines resulted Mycoplasma free by routine testing using Universal Mycoplasma detection kit (cat. No 30-1012K, ATCC).

### 4.6. Chemical Inhibitors

S3I-201 (NSC 74859, cat. No. S1155, Selleck Chemicals LLC, Houston, TX, USA) is a potent inhibitor of STAT3 protein dimerization with an IC50 of 86 μM and significantly lower affinity for STAT1 and STAT5 proteins [17]. S3I-201 was re-suspended in DMSO and used at the concentrations of 1, 10, 50, and 100 μM. Ruxolitinib (INCB018424, cat. No. S1378, Selleck Chemicals LLC) is a potent selective inhibitor of JAK1 and JAK2, approved FDA for clinical use, with an IC50 of 3.3 nM/2.8 nM (enzyme assay) and 130 times more selective for JAK1/2 than JAK3 [17]. The inhibitor was re-suspended in DMSO and used at the concentrations of 1 and 10 μM.

### 4.7. siRNA Delivery

STAT3 (Genbank accession n° NM_139276.2, NM_213662.1, NM_00315.3) knockdown was obtained using two STAT3-specific siRNA namely si-STAT3 (assay ID S744) and si2-STAT3 (assay ID S745). As control was used the control scrambled siRNA (ca. no. 4390846), obtained from Ambion (Thermo Fischer Scientific, Waltham, MA, USA). Lyophilized siRNAs were reconstituted according to manufacturer instructions, mixed with 1.5 µL of Lipofectamine^®^ 3000 Transfection Reagent (cat. No. L3000-015, Invitrogen™ for Thermo Fischer Scientific) in serum free Opti-MEM (cat. No. 31985-062, Gibco™) and delivered to the cells (50,000–70,000/well) at the final concentration of 5 nM in complete culture medium. For longer silencing, cells were exposed to a second dose of the same siRNA and incubated for additional 72 h. Efficiency and specificity of si-STAT3 was monitored by real-time reverse transcription-polymerase chain reaction (RT-PCR) and western blotting.

### 4.8. Western Blotting

The intracellular levels of pSTAT3, STAT3, FOSL1, MYC and actin proteins were determined by western blotting. Cells were washed, re-suspended in RIPA lysis buffer (cat. No. 89900, Pierce, Thermo Fischer Scientific) with a Protease Inhibitor Cocktail (cat. No. 78440, Sigma-Aldrich) and sodium orthovanadate (Na3VO4) (cat. No. 450243, Sigma-Aldrich), and kept in ice for 10 min. After 20 min centrifugation at 12,000 *g* at 4 °C, the supernatant was collected, and protein concentration determined by Bradford assay. A total of 20 μg of proteins were loaded on 4–12% NuPAGE^®^ Bis-Tris Mini Gels (cat. No. NP0335, Invitrogen™, Thermo Fisher Scientific) under reducing condition and transferred onto a PVDF membrane (cat. No. LC2007, Invitrogen™, Thermo Fisher Scientific). Membranes were incubated in the blocking solution 1% BSA (cat. No. A3059, Sigma-Aldrich) in T-TBS (TBS, 0.05% Tween 20) (cat. No. 28360, Invitrogen™, Thermo Fisher Scientific) for 1 h at room temperature; subsequently membrane was exposed to primary antibodies diluted in blocking solution, for 16 h at 4 °C. Primary antibodies are listed in Supplementary Table S12. After washing in TBS-T, the blots were incubated with the appropriate secondary antibodies (anti-Rabbit cat. no. sc-2077 Santa Cruz Biotechnology, Inc., Dallas, TX, USA, or anti-Mouse cat. No. 7076, Cell Signaling Technology Inc., Danvers, MA, USA), conjugated with horseradish peroxidase for 1 h at room temperature. Immunoreactive proteins were detected by SuperSignal™ West Pico Chemiluminescent Substrate (cat. No. 34577, Thermo Fisher Scientific), visualized by autoradiography.

### 4.9. Analysis of Cell Viability, Apoptosis and Proliferation

To evaluate cell viability, cells were counted for trypan blue exclusion using Countess^®^ II Automated Cell Counter (Invitrogen™, Thermo Fisher Scientific). Experiments were performed at 48 h or at day 6 after STAT3 inhibition by chemical inhibitors or genetic silencing respectively.

For cell death quantification, cells treated for 24 h using S3I-201 inhibitor were trypsinized, washed and re-suspended in complete medium. Cell apoptosis was investigated by Pacific Blue™ Annexin V/SYTOX™ AADvanced™ Apoptosis Kit, for flow cytometry (cat. No. A35136, Thermo Fisher Scientific), following manufacturer’s instructions. Pacific Blue™ Annexin V/SYTOX™ AADvanced™ emissions were analyzed by flow cytometry using a MACS Quant Analyzer (Miltenyi Biotec GmbH, Bergisch Gladbach, Germany). Data were analyzed using FlowJo, LLC (Ashland, OR, USA).

For cell proliferation using BrdU incorporation, cells were plated on glass in complete medium. After starvation in 0.5% FBS containing medium for 24 h, were indicated cells were pre-treated with 50 µM S3I-201 or 1 µM RX for 2 h and then stimulated with IL6 (100 ng/mL, cat. No. 200-06, Peprotech) for 36 h. Non stimulated cells were cultured in 0.5% FBS containing medium. For BrdU incorporation, cells were pulsed with 100 µM BrdU (Sigma-Aldrich) for 30 min at 37 °C. Cells were washed twice and fixed in cold 70% ethanol for 5 min at room temperature. After washing, cells were incubated with 1.5 M HCl for 30 min at room temperature. Then, cells were washed and incubated in PBS containing 1% FBS (blocking buffer) for 60 min and incubated with anti-BrdU (Bu20a, cat. No. 5292, Cell Signaling Technology, diluted 1:1000) for 16 h at 4 °C. After washing, cells were incubated with goat anti-mouse IgG F(ab’)2 fragment Texas Red-conjugated (cat. No. SAB3701138, diluted 1:500, Sigma-Aldrich) for 30 min at room temperature. Cells were washed in PBS several times and mounted with ProLong Gold antifade with DAPI (cat. No. D3571 Invitrogen™). Fluorescence images were captured by using a Leica DM5000 B microscope. Image analysis was performed by using ImageJ software. For cell proliferation after cell silencing, mock and silenced-UBC cells (10,000–40,000/well) were plated in 96-well plates and allowed to adhere overnight in 10% FBS complete medium. The next day, the medium was replaced with fresh complete medium and cells were cultured for five days. For the colony formation assay, cells were fixed with 4% PFA for 15 min and stained with 0.1% crystal violet for 10 min and wash with MQ water; dried cells were incubated with 10% acetic acid to solubilize the crystal violet. Absorbance was read at 590 nm. Alternatively, cell viability was evaluated using the CellTiter 96^®^ AQueous One Solution Cell Proliferation Assay, contains the 3-(4,5-dimethylthiazol-2-yl)-5-(3-carboxymethoxyphenyl)-2-(4-sulfophenyl)-2H-tetrazolium, inner salt, MTS (ca. No. G3582, Promega), according to the manufacturer’s instructions.

### 4.10. Quantitative RT-PCR (qRT-PCR)

mRNAs targets were quantified by reverse transcription-polymerase chain reaction (qRT-PCR) assay using the StepOnePlus™ Real-Time PCR System (Applied Biosystems, Thermo Fisher Scientific). Total RNA was extracted from MIBCs cells by TRIzol™ (15596026, Invitrogen™, Thermo Fisher Scientific). The cDNAs were synthesized by SuperScript^®^ VILO™ Master Mix (11755, Invitrogen™, Thermo Fisher Scientific) from 1 µg of total RNA, in a total volume of 20 µL. One µL of the cDNA synthesis reaction was used for the specific amplification of the target transcript and of the Hypoxanthine-guanine phosphoribosyltransferase 1 (HPRT1), as normalization control. The PCR was performed in a total volume of 20 µL at 50 °C for 2 min, 95 °C for 10 min, followed by 40 cycles at 95 °C for 15 s and at 60°C for 1 min, with TaqMan^®^ Universal Master Mix II (cat. No. 4369016, Applied Biosystems, Thermo Fisher Scientific) and the TaqMan™ Gene Expression Assays (Applied Biosystems, Thermo Fisher Scientific) (Supplementary Table S5). The threshold cycle (CT) was determined for each sample in duplicates and quantification was performed using the comparative CT method (∆∆CT).

### 4.11. Invasion Assay

Polycarbonate transwell fiters (8 μm pore size, cat. no 3422, Corning, NY, USA) were coated with 50 μg of reduced growth factor basement membrane extract (RGF BME; 10 mg/mL; cat. no. 3433-005-01, Cultrex) diluted in a total volume of 150 μL of serum-free medium. Then the transwells were placed in a 24 well/plate. Twenty-four hours STAT3 silenced 5637 and RT4 cells, or RX and S3I-201 treated cells, as well as not treated cells, were serum starved for 24 h, trypsinized, resuspended in 150 μL of serum-free medium and seeded into the coated filter at a concentration of 100 cells/well. 600 μL of complete medium were added into the lower chamber to take advantage of FBS as chemo-attractant factor. The plate was incubated at 37 °C and allowed to migrate through the matrigel-coated filter. After 24 h of incubation, cells that had crossed the filter were fixed, stained with crystal violet and counted.

### 4.12. Data Preprocessing and Statistical Analysis of the TCGA and GSE32894 Datasets

STAT3 and FOSL1 gene signature were obtained by literature review (Pubmed search). Raw counts for primary solid tumor samples were downloaded from GDC portal harmonized repository using TCGAbiolinks R/Bioconductor package (N = 408 cases). The FFPE samples were removed and only samples corresponding to Luminal or Basal subtype were kept. The duplicated samples counts were averaged (N = 388). The library size normalization factors were obtained with the trimmed mean of M-values (TMM) [48] and gene expression was computed as log2-RPKM. For the construction of a classifier for UBC subtype we randomly split data in two sets, Training and Test sets, with a balanced ratio for the outcome variable using a 70–30% proportion. Classification was performed using regularized logistic regression via elastic-net algorithm. Model parameters (alpha and lambda) were jointly tuned using 10-fold cross-validation. Model performances were reported as classification accuracy. For all the classification procedure we also tried to identify a more stable subset of genes using a stability path algorithm, with threshold 80% and per-comparison error rate of 10%.

For the purpose of building a survival prediction model data were split in a training and test set trying to achieve a similar mortality rate in both groups. Prediction for OS was performed using regularized Cox proportional hazards regression models applying the elastic-net algorithm with regularization parameters tuning based with 10-fold cross-validation. We also implemented a modified version of the Bolasso algorithm. Briefly, bootstrap samples of the training set (B = 200) were drawn and Lasso variable selection was performed on each random subset. Genes selected (beta not equal to zero) in at least 50% of the random samples were retained. These features were used in a ridge regularized model defined “Boot Lasso”.

To evaluate the predictive accuracy of these models we calculated prediction errors over time using the Integrated Brier Score. Accuracy of the predictions were compared using 10-fold cross-validation.

The bivariate distribution of survival times and signatures values can be computed using a nearest-neighbors estimator using a rectangular kernel. For descriptive purposes survival curves as a function of signature value were plotted using conditional Kaplan-Meir estimator. For illustration, we show the estimated survival curves for the neighborhood of the smallest, median, and largest value of the signature, respectively labelled as low, medium and high. Simply put, the three curves represent the survival estimates for a fraction of the samples with the closest values to the smallest, median and largest signature values respectively.

NMIBC were defined as tumor stage T0–T1 while MIBC were stage T2–T4. We evaluated the predicted performance of FOSL1 signature through repeated 10-fold cross validated logistic models with elastic-net regularization.

To evaluate Basal/Luminal subtype classification performance on an independent data set (GSE32894) we first merged data expression and adjusted for batch effect using the ComBat algorithm [49]. We then fitted a regularized logistic regression model with the same tuning parameters on the training set and evaluated the prediction accuracy on the external data. We used maximally selected log-rank statistics to test for a possible cut point with respect to predictor levels.

Gene differential expression was evaluated using linear modelling “limma” [50] performed on read counts transformed to log-CPM values. Observational-level weights obtained from the voom [51] function were used to model mean-variance relationship.

### 4.13. Statistical Analysis

For in vitro experiments MIBC cell lines were analyzed using the Student’s *t*-test. The One-way ANOVA with Bonferroni’s correction was used for multiple comparisons. Statistical significance was defined at a 5% significance level (*p* < 0.05). The analysis of the expression of STAT3-regulated transcripts was performed by using a Student *t*-test with False Discovery Rate correction (FDR ≤ 0.05). For histological, clinical and pathological analysis of human BC, the qualitative variables were described as absolute and relative frequencies.

## 5. Conclusions

Data obtained from this study suggest that monitoring pSTAT3 and the downstream targets, particularly FOSL1, could provide meaningful level of UBC stratification. Moreover, blockade of the STAT3 pathway might represent a novel treatment option for a subset of UBC patients.

## Supplementary Materials

The following are available online at https://www.mdpi.com/2072-6694/11/9/1219/s1, Supplementary File 1: Figure S1: Nuclear pSTAT3 expression in UBC biopsies; Figure S2: pSTAT3 staining in the tumor microenvironment of UBC; Figure S3: Higher magnification of cases reported in the main Figure 2 illustrating the basal phenotype of pSTAT3 expressing cells in MIBC (Digital resized); Figure S4: pSTAT3 expression in the microenvironment of human basal-type MIBC; Figure S5: UBC cell lines growth curves (RT4, 5637) and knockdown of STAT3 in UBC cell lines (5637, HT-1376, RT4 and T24); Figure S6: Effects of STAT3 silencing on proliferation of UBC cell lines monitored by Crystal violet assay and MTS assay; Figure S7: Effect of S3I-201 and RX on STAT3 phosphorylation; Figure S8: Effects of STAT3 inhibition on the apoptosis of UBC cell lines; Figure S9: Effects of STAT3 inhibition on proliferation of UBC cell lines monitored by BrdU incorporation; Figure S10: Effects of S3I-201 on UBC cells invasion; Figure S11: Effect of JAK1 silencing on STAT3 phosphorylation; Figure S12: Histograms of class probability for STAT3 signature (TCGA dataset); Figure S13: Prediction Error Curves for STAT3 signature (TCGA dataset); Figure S14: Test for cut-point for STAT3 signature (TCGA dataset); Figure S15: Effect of STAT3 inhibition on MYC and FOSL1 protein expression; Figure S16: Prediction error curves for FOSL1 signature (Boot Lasso model) both for training (A) and test set (B) (TGCA dataset); Figure S17: Kaplan-Meier estimate of the dichotomized FOSL1 signature (TCGA dataset); Figure S18: Controls for pSTAT3 and FOSL1 IHC; Figure S19: Whole western-blots corresponding to Supplementary Figure S5A, Figure S7B, Figure S11C and Figure S15D and densitomentric quantification of each bands relative to actin control bands. Supplementary File 2: Table S1: Clinical and pathological features of UBC patients and correlation with pSTAT3 expression; Table S2: Patients characteristics of MIBCs (retrospective UBC cohort); Table S3: MIBC subtype by immunohistochemistry score (retrospective UBC cohort); Table S4: Patients characteristics and pSTAT3 score of NMIBCs (retrospective UBC cohort); Table S5: TaqMan™ Gene Expression Assays; Table S6: Fold Change analysis of the expression of STAT3-regulated transcripts in MIBC cell lines; Table S7: Coefficients for UBC subtype (Basal, Luminal) classification using elastic-net for STAT3 targets (TCGA cohort); Table S8: Hazard Ratios (HR) for univariate Cox models testing overall survival in STAT3 signature TCGA dataset; Table S9: Fold Change (FC) for Luminal vs. Basal comparison in FOSL1 signature in TCGA and GSE32894 datasets; Table S10: Fold change (FC) for MIBCs vs. NMIBCs comparison in FOSL1 signature in GSE32894 dataset; Table S11: Coefficients for MIBCs vs. NMIBCs classification using FOSL1 signature in GSE32894 dataset; Table S12: IHC and western blotting antibodies.
