# Supplementary material for: Hyper-Activation of STAT3 Sustains Progression of Non-Papillary Basal-Type Bladder Cancer via FOSL1 Regulome
Source: Cancers (Basel). 2019 Aug 21;11(9):1219. doi: 10.3390/cancers11091219 (PMC6770563; doi:10.3390/cancers11091219)
Supplement: Supplementary file 1 [file cancers-11-01219-s001.zip › cancers-565379-supplementary/Benerini Gatta et al. Supplementary File 1.pdf]

Supplementary File 1

Hyper-activation of STAT3 sustains progression of non-papillary basal-type bladder cancer via FOSL1 regulome

Luisa Benerini Gatta [1,,2,3,†], Laura Melocchi [4,†], Mattia Bugatti [1,3], Francesco Missale [5], Silvia Lonardi [1,3], Benedetta Zanetti [1,3], Luca Cristinelli [2,3], Sandra Belotti [2,3], Claudio Simeone [2,3], Roberto Ronca [1], Elisabetta Grillo [1], Sara Licini [1,3], Debora Bresciani [1,3], Regina Tardanico [1,3], Szeman Ruby Chan [6], Emanuele Giurisato [7,8], Stefano Calza [1,†] and William Vermi [1,3,9,*]

Supplementary figures

Supplementary Figure S1 shows the nuclear expression of pSTAT3 in UBC IHC scoring system.

Supplementary Figure S2 shows pSTAT3 staining in the microenvironment of UBC.

Supplementary Figure S3 illustrates the higher magnification of main Figure 2.

Supplementary Figure S4 illustrates pSTAT3 expression in the microenvironment of human basal-type MIBC.

Supplementary Figure S5 illustrates the growth curve of UBC cells and the efficiency of STAT3 knockdown of si-STAT3 and si2-STAT3 in UBC cell lines.

Supplementary Figure S6 shows the effects of STAT3 inhibition on proliferation of UBC cell lines monitored by Crystal violet assay and MTS assay.

Supplementary Figure S7 shows the effect of S3I-201 and RX on STAT3 phosphorylation.

Supplementary Figure S8 shows the effects of STAT3 inhibition on the apoptosis of UBC cell lines.

Supplementary Figure S9 shows the effects of STAT3 inhibition on tumor cell proliferation of UBC cell lines monitored by BrdU incorporation after IL6 stimulation.

Supplementary Figure S10 shows the effect of S3I-201 on UBC cells invasion.

Supplementary Figure S11 shows the effect of JAK1 silencing on STAT3 phosphorylation.

Supplementary Figure S12 shows the histograms of class probability for STAT3 signature (TCGA dataset).

Supplementary Figure S13 illustrates the prediction error curves for STAT3 signature (TCGA dataset).

Supplementary Figure S14 illustrates the test for cut point for STAT3 signature (TCGA dataset).

Supplementary Figure S15 shows the effect of STAT3 inhibition on MYC and FOSL1 protein expression.

Supplementary Figure S16 illustrates the prediction error curves for FOSL1 signature (TCGA dataset).

Supplementary Figure S17 illustrates the Kaplan-Meier estimate of the dichotomized FOSL1 signature (TCGA dataset).

Supplementary Figure S18 illustrates control stains for pSTAT3 and FOSL1.

Supplementary Figure S19 illustrates the whole western-blots corresponding to Supplementary Figures S5, S7, S11, S15 and densitometric quantification of each bands relative to actin control bands.

FIGURE S1

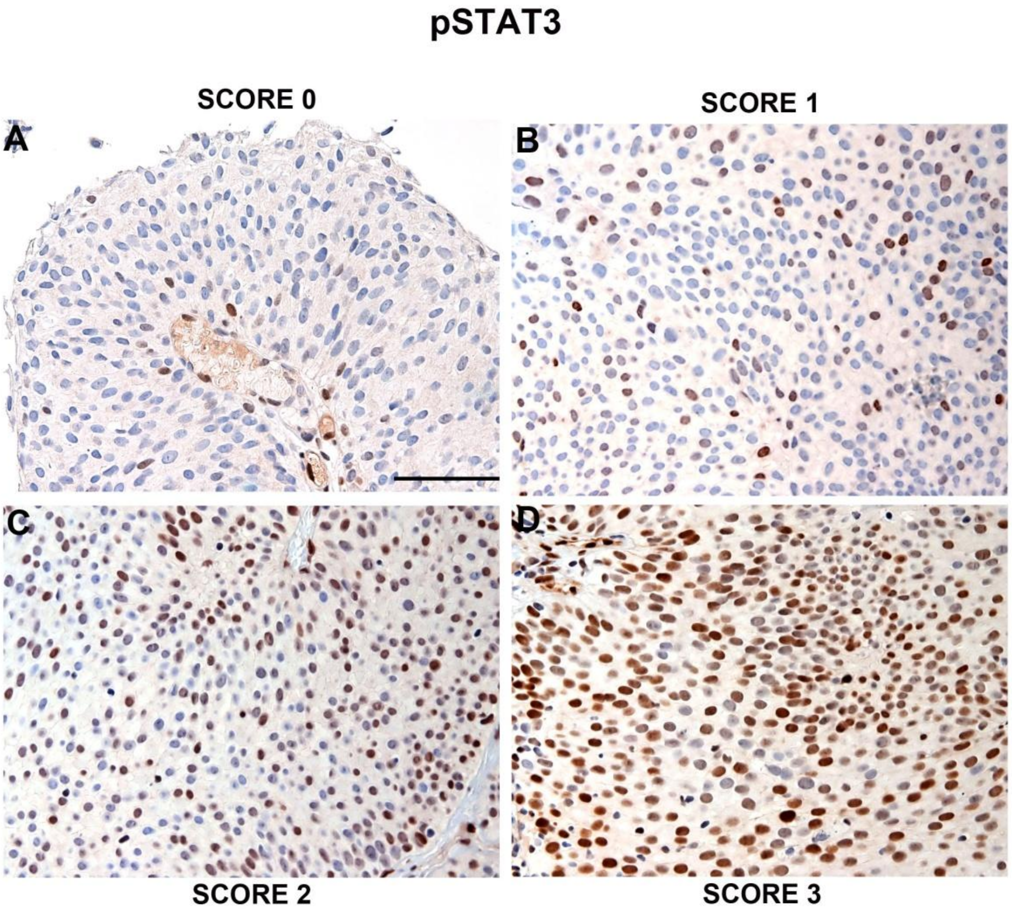

Supplementary Figure S1. Nuclear pSTAT3 expression in UBC biopsies. Examples of pSTAT3 scoring system: A) Score 0 = < 5 % of positive cells B) Score 1 = < 25 % of positive cells C) Score 2 = 25-50 % of positive cells D) Score 3 = > 50 % of positive cells. Magnification 200X. Scale bar = 100 μm.

FIGURE S2

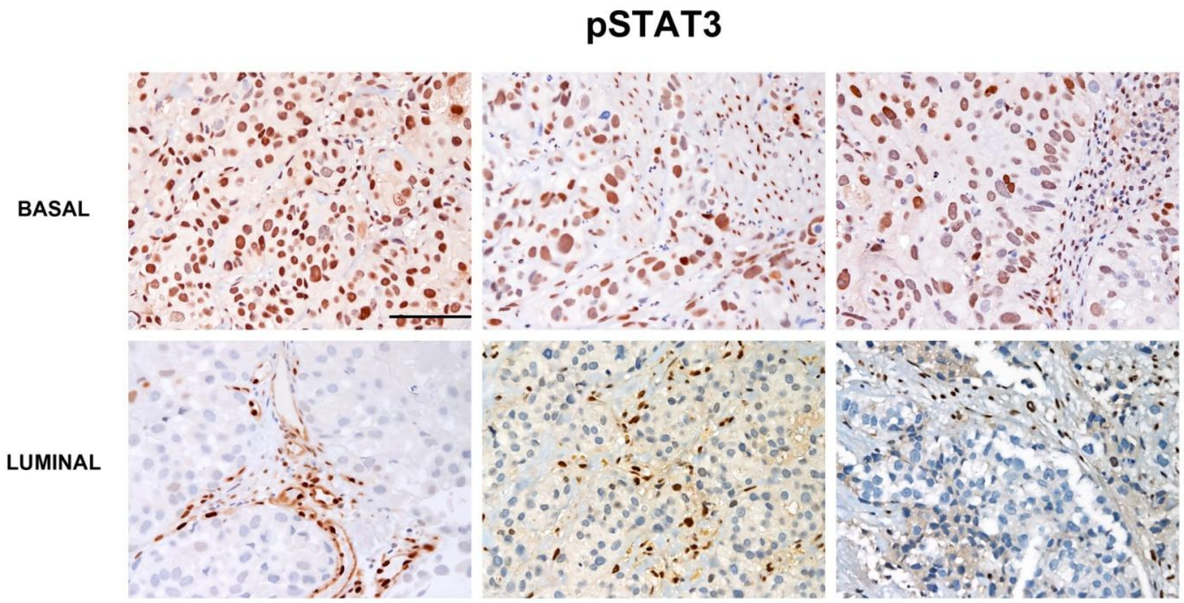

Supplementary Figure S2. pSTAT3 staining in the tumor microenvironment of UBC. Panels are from cases of basal-type (n = 3) and luminal-type (n = 3) MIBCs stained for pSTAT3. pSTAT3 stains neoplastic cells and that of tumor microenvironment in basal-type (upper panels), but only cells of microenvironment in luminal-type (bottom panels). Magnification 200X. Scale bar = 100 μm.

FIGURE S3

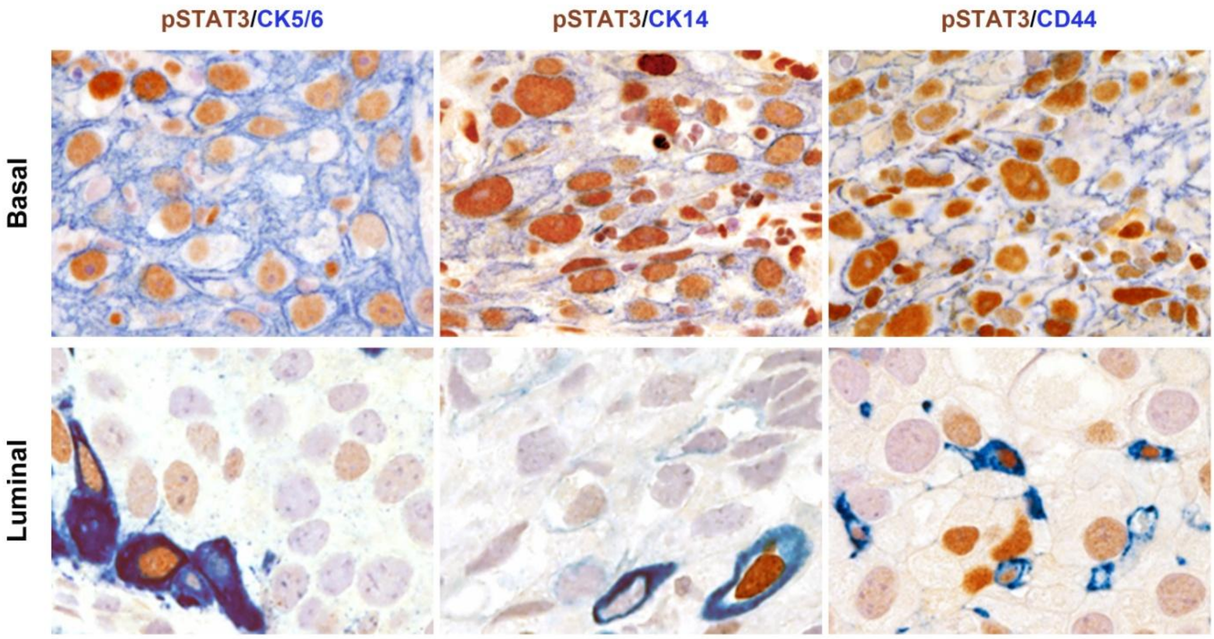

Supplementary Figure S3. Higher magnification of cases reported in the main Figure 2 illustrating the basal phenotype of pSTAT3 expressing cells in MIBC (Digital resized).

FIGURE S4

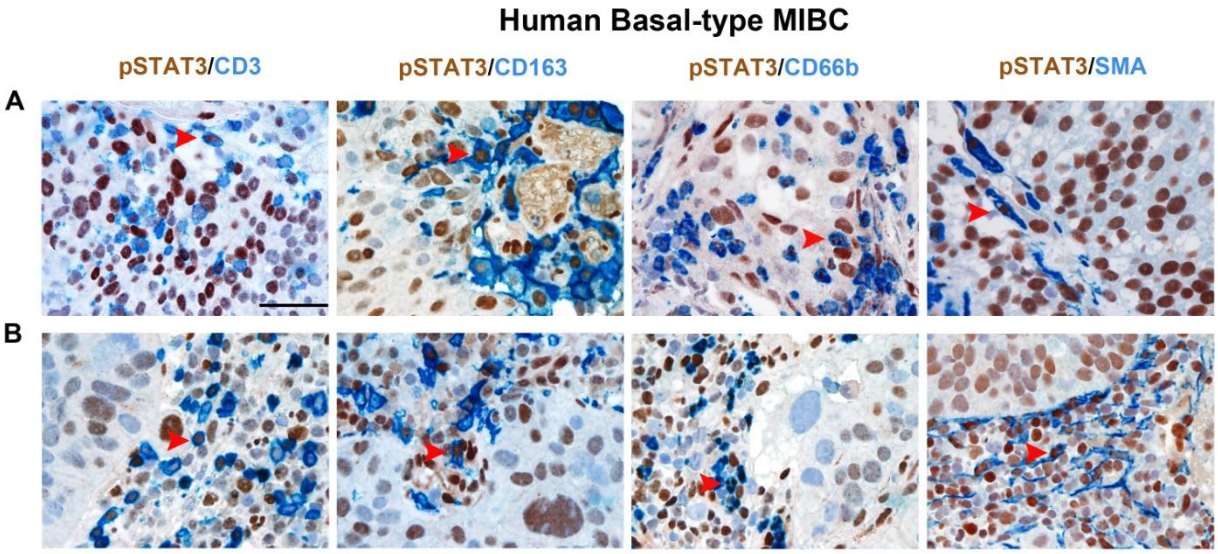

Supplementary Figure S4. pSTAT3 expression in the microenvironment of human basal-type MIBC. Magnification 400X. Scale bar = 50 μm.

FIGURE S5

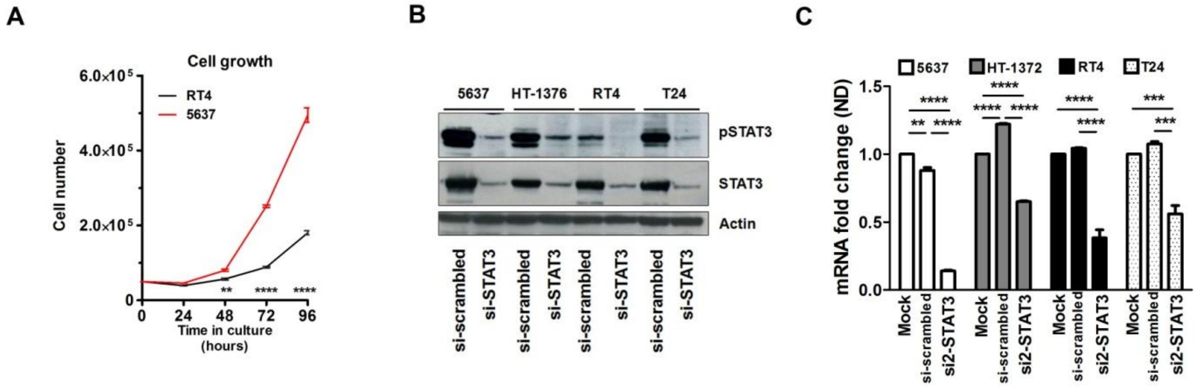

Supplementary Figure S5. UBC cell lines growth curves (RT4 and 5637) and knockdown of STAT3 in UBC cell lines (5637, HT-1376, RT4 and T24). In A the graph shows the growth curve of basal-type 5637 cells vs luminal-type RT4 cells at 24, 48, 72, and 96 hours. Two way ANOVA test was used for statistical analysis (**$p < 0.01$, ****$p < 0.0001$). In B, the image shows a representative western blotting of the pSTAT3/STAT3 proteins reduction after 5 nM si-STAT3 knockdown for 144 hours. The actin level is for control loading gel. In C, the graph shows the efficiency of 5 nM si2-STAT3 knockdown in UBC cell lines. One way ANOVA test was used for statistical analysis (**$p < 0.01$, ***$p < 0.001$, ****$p < 0.0001$).

FIGURE S6

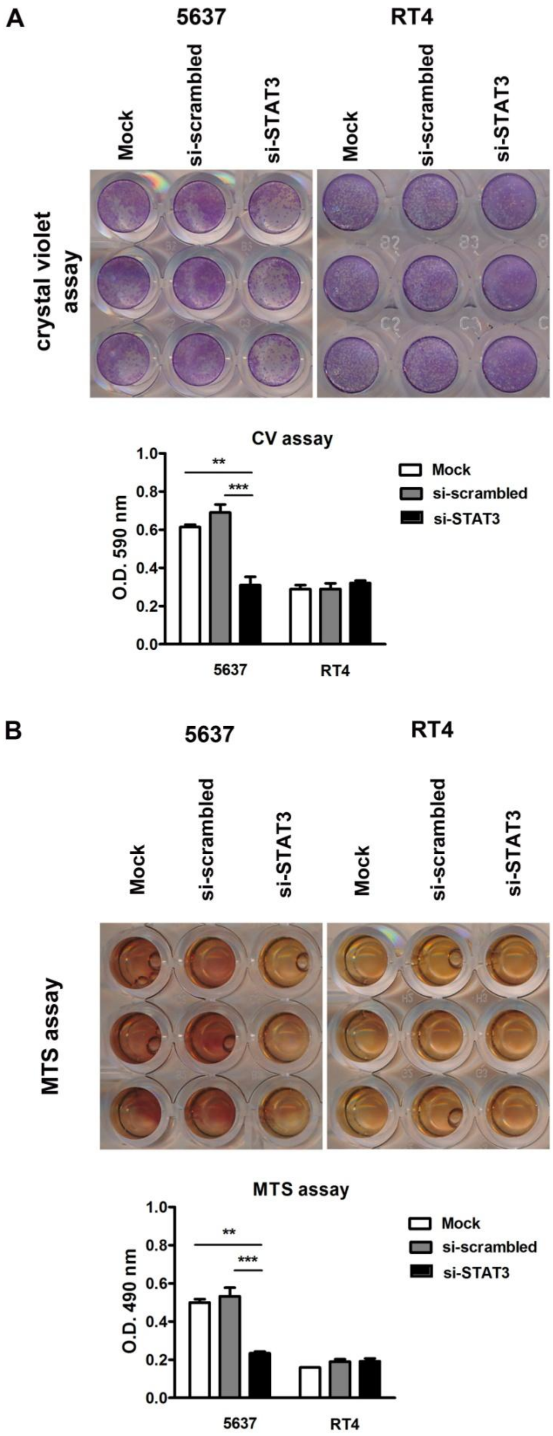

Supplementary Figure S6. Effects of STAT3 silencing on proliferation of UBC cell lines monitored by Crystal violet assay and MTS assay. Representative images of the basal-type 5637 cell line and the luminal-type RT4 cell line using the Crystal violet assay (A) or MTS assay (B). Silenced-UBC cells (10000-40000/well) were plated and proliferation monitored for 5 days. The graphs show the mean ± S.E.M. of the staining in triplicate (n = 2). The One-way ANOVA test was used for statistical analysis (**p < 0.01***, p < 0.001).

FIGURE S7

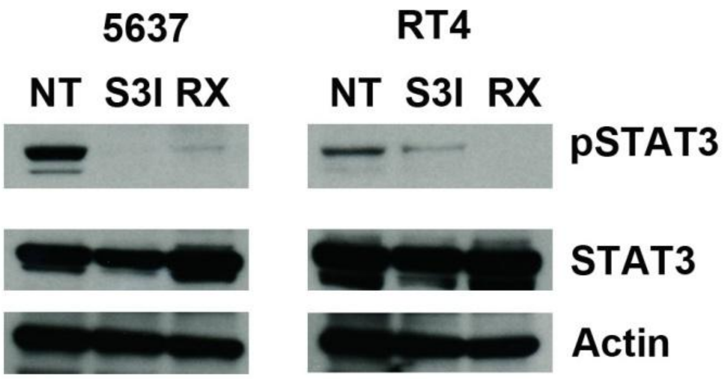

Supplementary Figure S7. Effect of S3I-201 and RX on STAT3 phosphorylation. The image shows a representative western blotting of the pSTAT3/STAT3 proteins reduction after 24 hours of treatment with the drugs S3I-201 100 μM and RX 1 μM in 5637 (A) and RT4 (B) cells. The Actin level is for control loading gel. NT= not-treated.

FIGURE S8

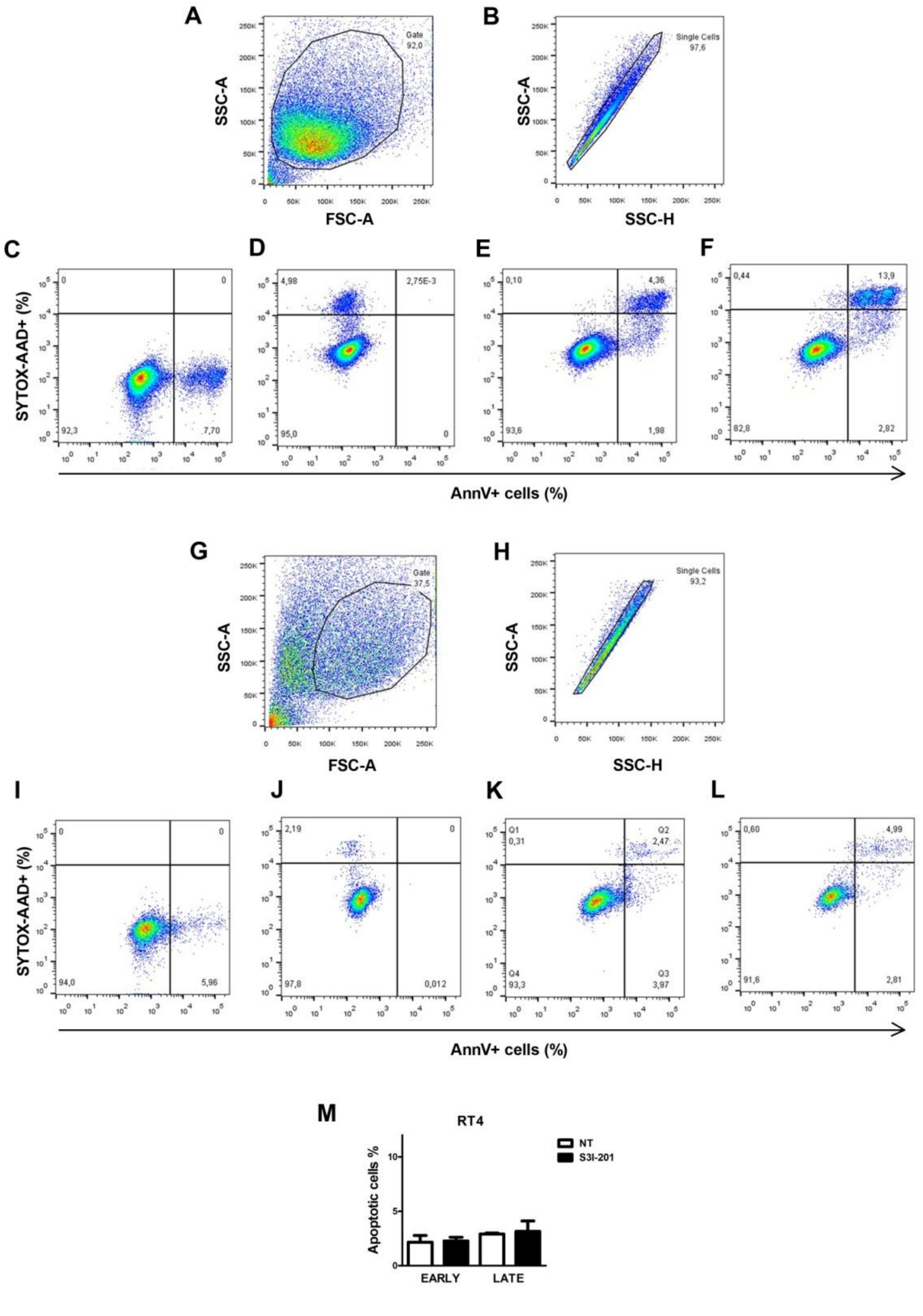

Supplementary Figure S8. Effects of STAT3 inhibition on the apoptosis of UBC cell lines. The flow cytometry plots illustrate the gating strategy (A-D, G-J) used to identify AnnV+ cells (C,I) and SYTOX-AAD+ cells (D,J) in basal-type 5637 cells and luminal-type RT4 cells respectively. Representative plots illustrating the cell percentages of apoptotic cells in control cells (E, K) and S3I-201 treated cells (F, L) for 24 hours. The graph (M) shows the Means with S.E.M. of three representative experiments using RT4 cells. The One-way ANOVA test was used for statistical analysis

FIGURE S9

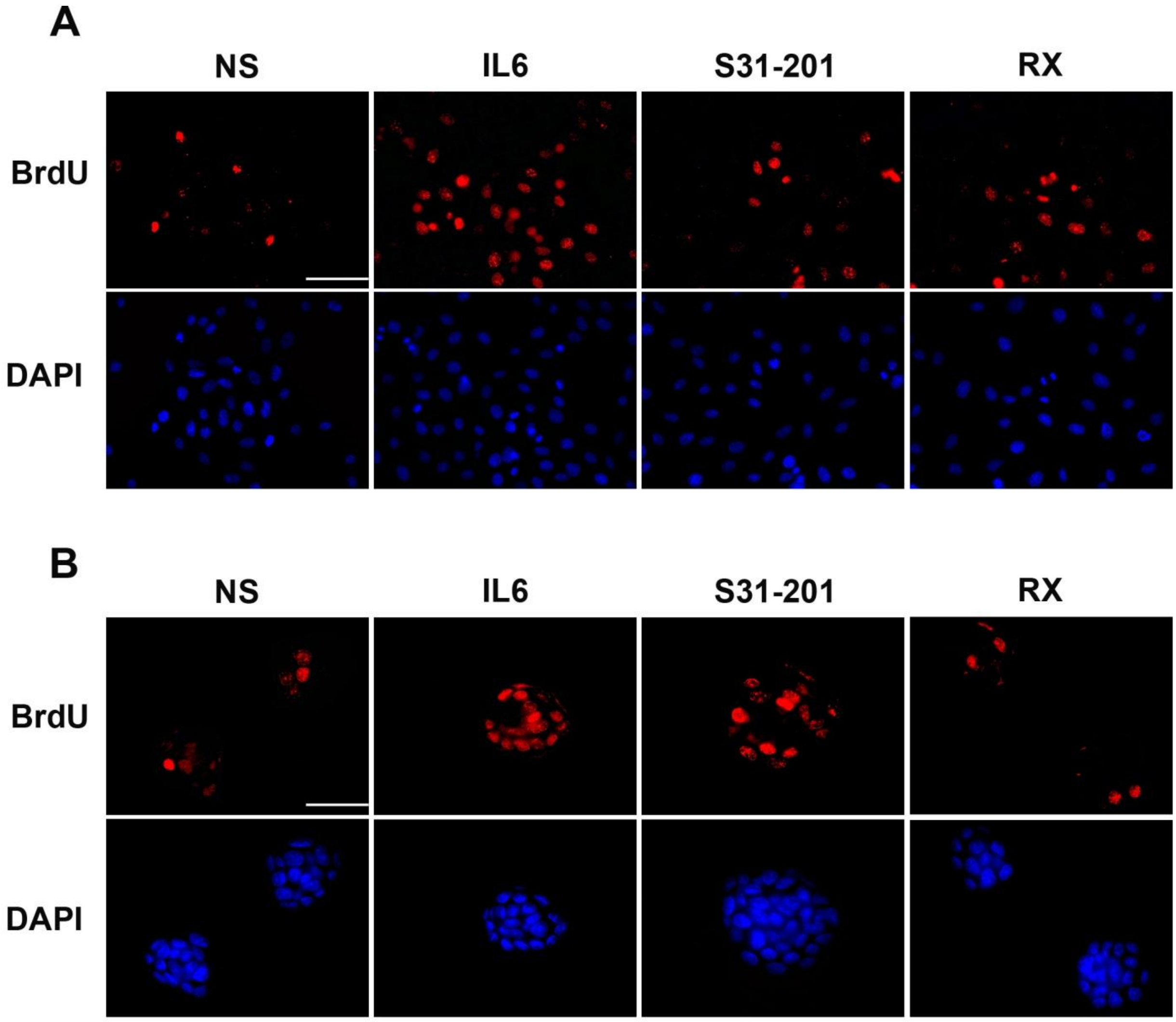

Supplementary Figure S9. Effects of STAT3 inhibition on tumor cell proliferation of UBC cell lines monitored by BrdU incorporation. Representative immunofluorescence staining of basal-type 5637 (A) and luminal-type RT4 (B) using a specific antibody to BrdU (Red). DNA was stained with DAPI (blue). Magnification 400X. Scale bar = 50 µm. NS = Not-stimulated.

FIGURE S10

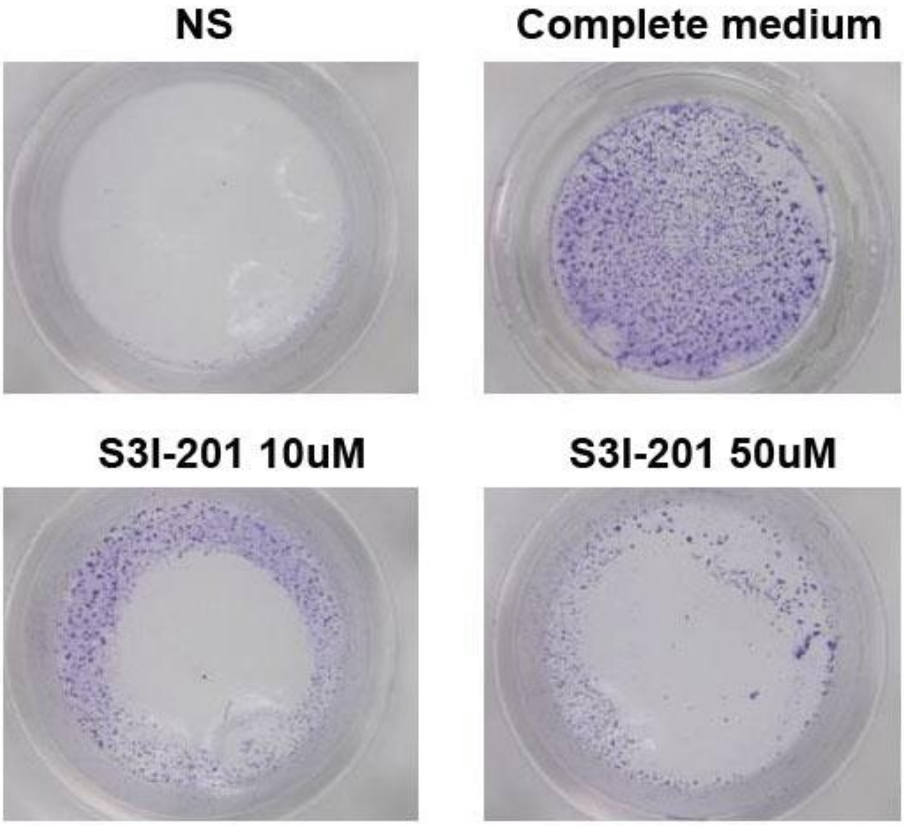

Supplementary Figure S10. Effects of S3I-201 on UBC cells invasion. The image shows a representative experiment of the reduction of 5637 cell invasion in a transwell after 24 hours of treatment using 10-50 μM of S3I-201 as labelled. NS = not-stimulated.

FIGURE S11

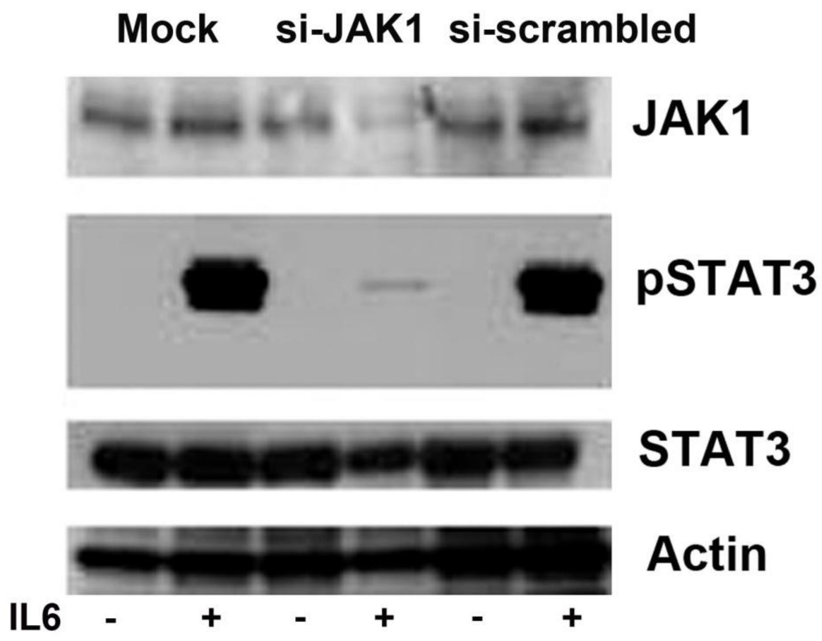

Supplementary Figure S11. Effect of JAK1 silencing on STAT3 phosphorylation. The image shows a representative western blotting of the pSTAT3/STAT3 proteins reduction after 5 nM si-JAK1 knockdown and IL6 stimulation (100 ng/ml) for 15 minutes in 5637 starved cells. The JAK1 protein level is for control of the silencing. The actin protein level is the loading control.

FIGURE S12

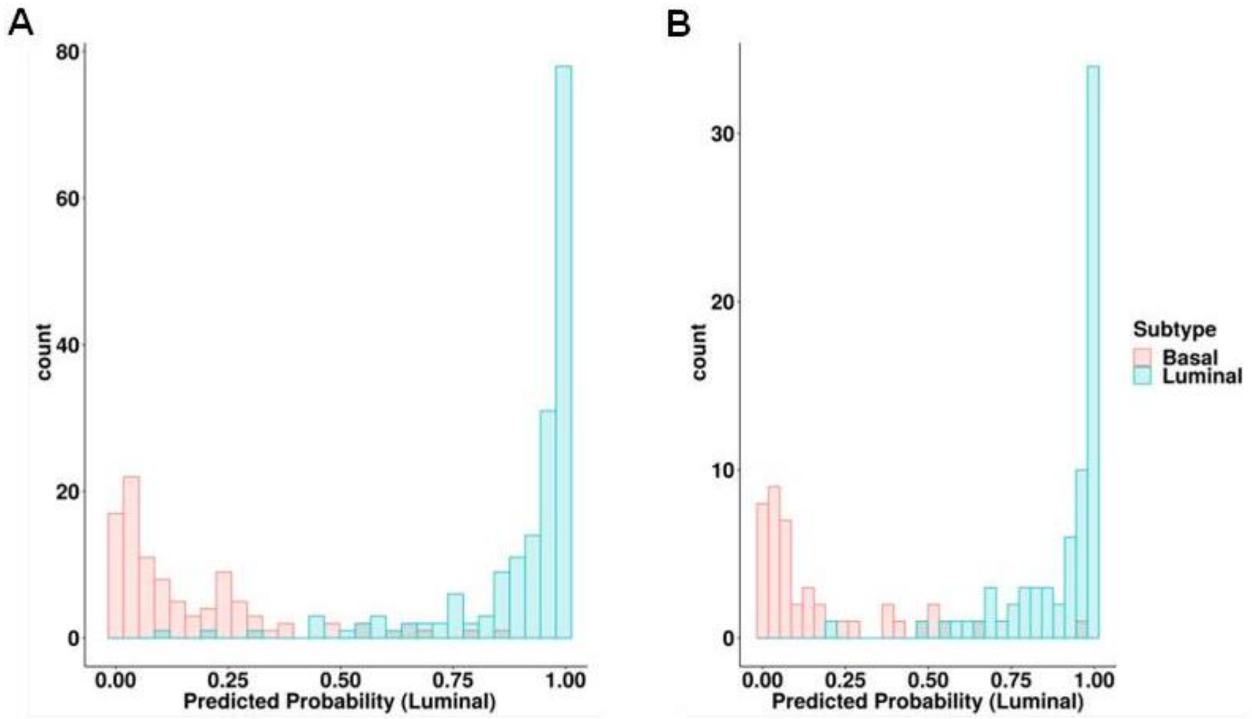

Supplementary Figure S12. Histograms of class probability for STAT3 signature (TCGA dataset). Histograms illustrate the distribution of predicted probability of TCGA samples belonging to class Luminal as estimated by regularized logistic model. A) Training set B) Test set. A clear separation is evident in the training set and is confirmed in the test set, suggesting a predicted role of the selected STAT3 signature.

FIGURE S13

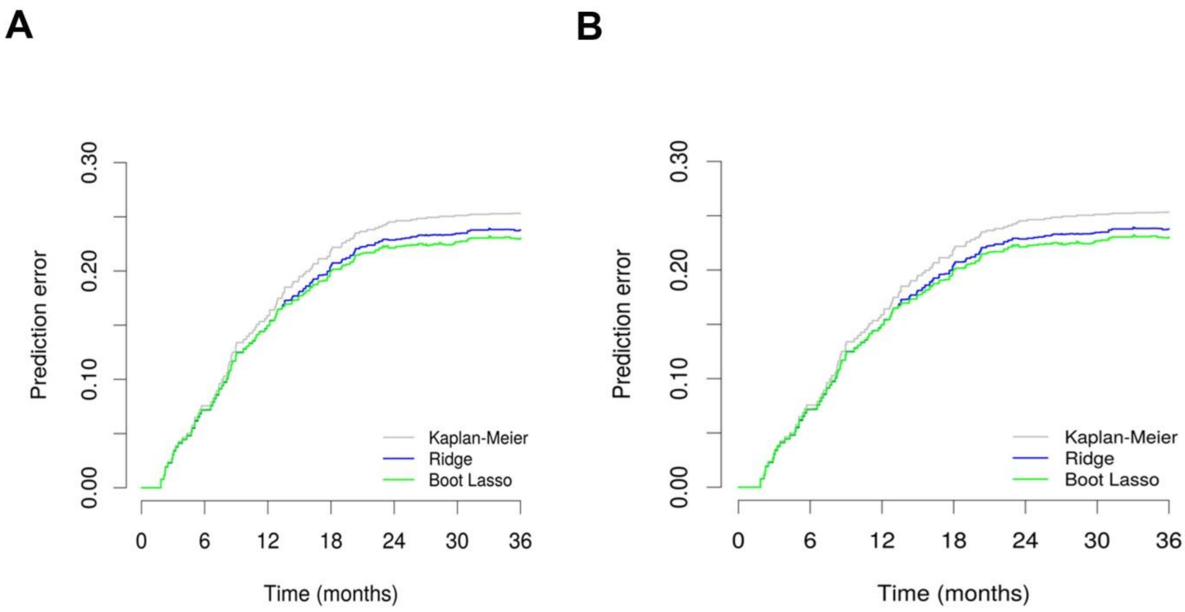

Supplementary Figure S13. Prediction Error Curves for STAT3 signature (TCGA dataset). The plots show the prediction error curves (PEC) for Overall Survival for the different feature selection procedures described in the text including Ridge (all genes) and Boot Lasso (subset). Grey curve represents a "null" model with no genetic information (Kaplan-Meier predictor). A) Training set: cross-validated PEC B) Test set.

FIGURE S14

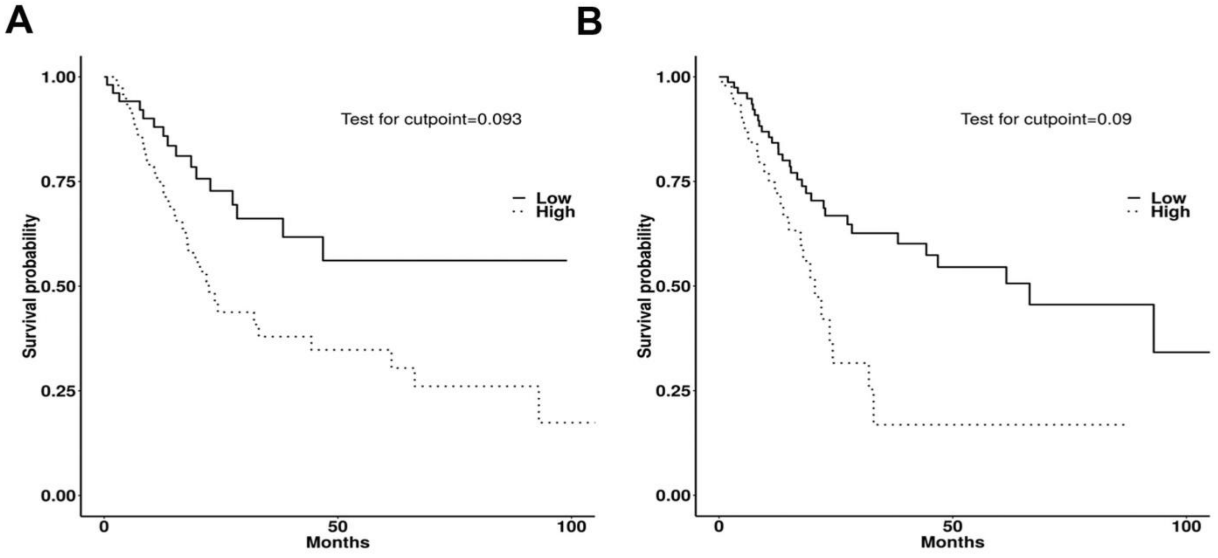

Supplementary Figure S14. Test for cut-point for STAT3 signature (TCGA dataset). Kaplan-Meier estimate of the dichotomized STAT3 signatures. The cut-point for dichotomization was determined based on maximally selected log-rank statistics. A) Full signature (Ridge) B) “Boot Lasso” subset.

FIGURE S15

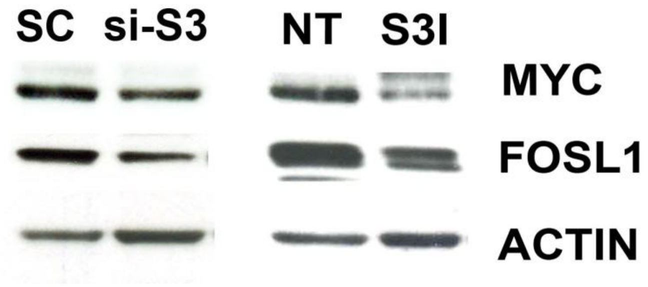

Supplementary Figure S15. Effect of STAT3 inhibition on MYC and FOSL1 protein expression. The image shows a representative western blotting of the MYC and FOSL1 protein reduction after 144 hours of STAT3 knockdown by siRNA STAT3 (si-S3) or 24 hours of treatment with S3I-201 (100 μM) in 5637 cells. The actin level is used as loading control. NT= not-treated cells.

FIGURE S16

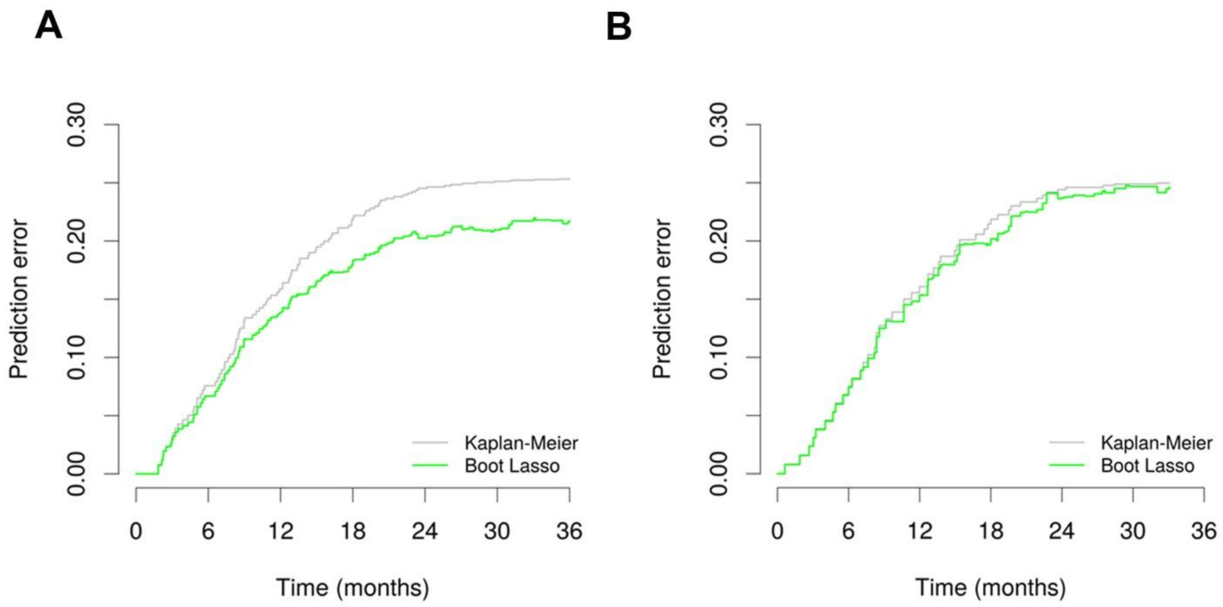

Supplementary Figure S16. Prediction error curves for FOSL1 signature (Boot Lasso model) both for training (A) and test set (B) (TGCA dataset).

FIGURE S17

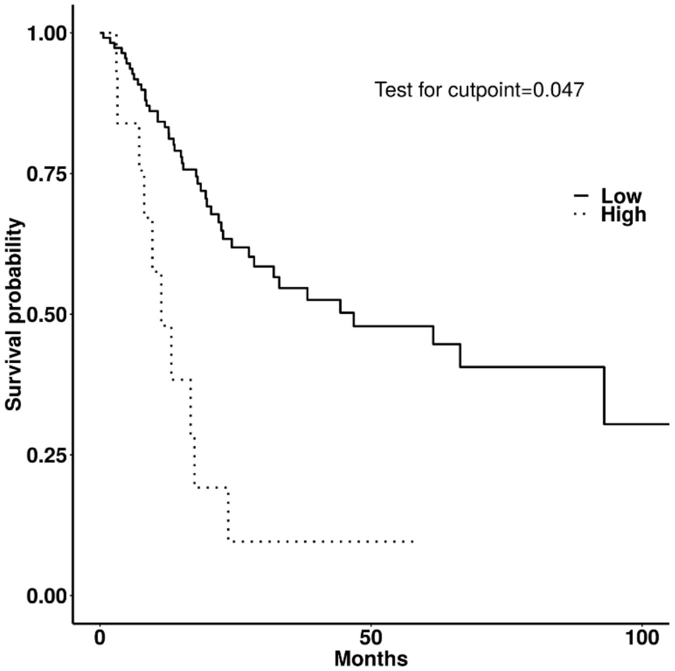

Supplementary Figure S17. Kaplan-Meier estimate of the dichotomized FOSL1 signature (TCGA dataset). The cut-point for dichotomization was determined based on maximally selected log-rank statistics.

FIGURE S18

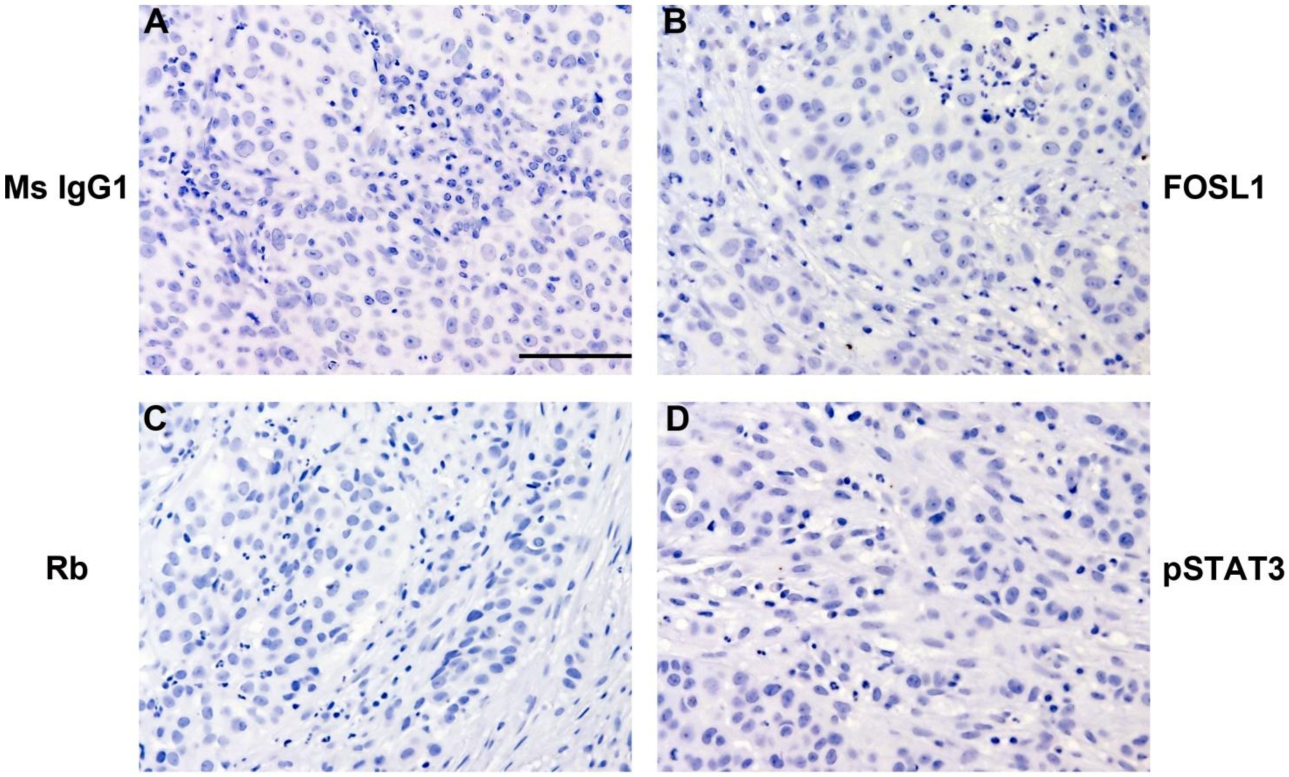

Supplementary Figure S18. Controls for pSTAT3 and FOSL1 immunohistochemistry. Isotype control (A, C) and omission of the primary antibody (B, D) for FOSL1 (A, B) and pSTAT3 (C, D). Ms = Mouse; Rb = Rabbit.

FIGURE S19

A

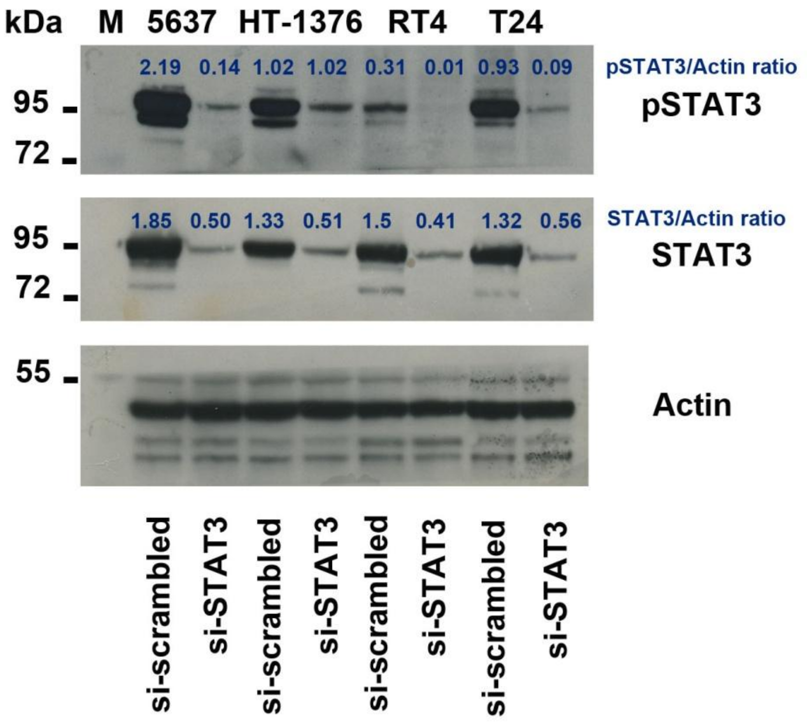

B

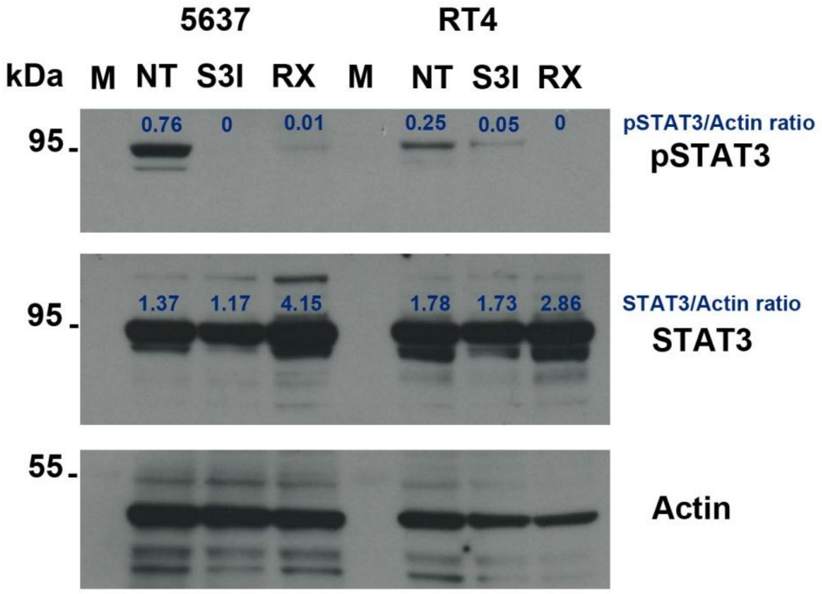

Figure S19 *cont.*

C

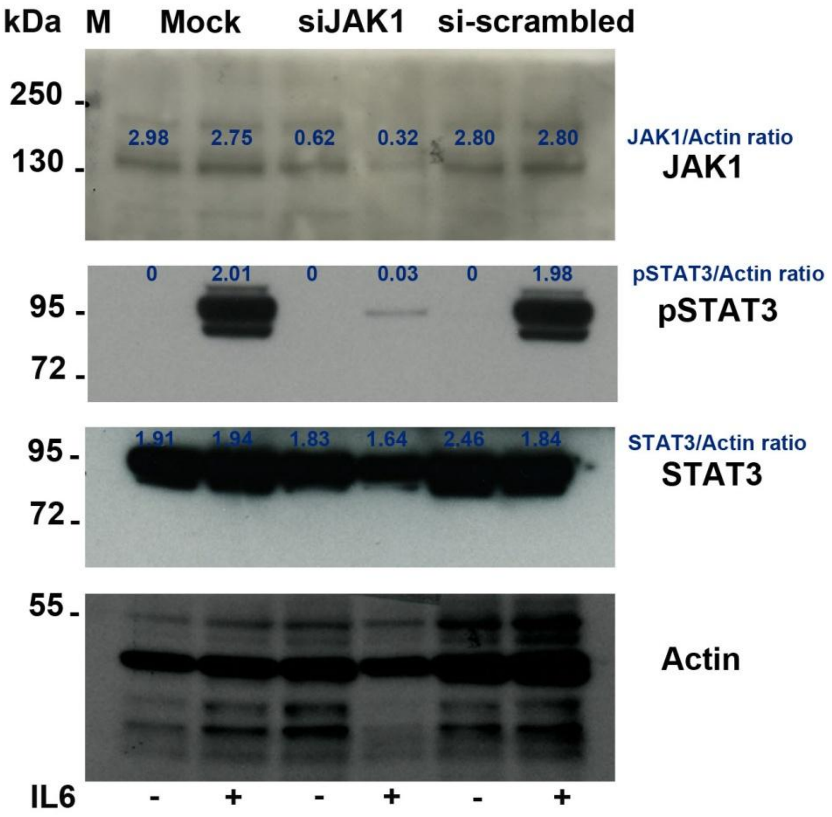

Figure S19 *cont.*

D

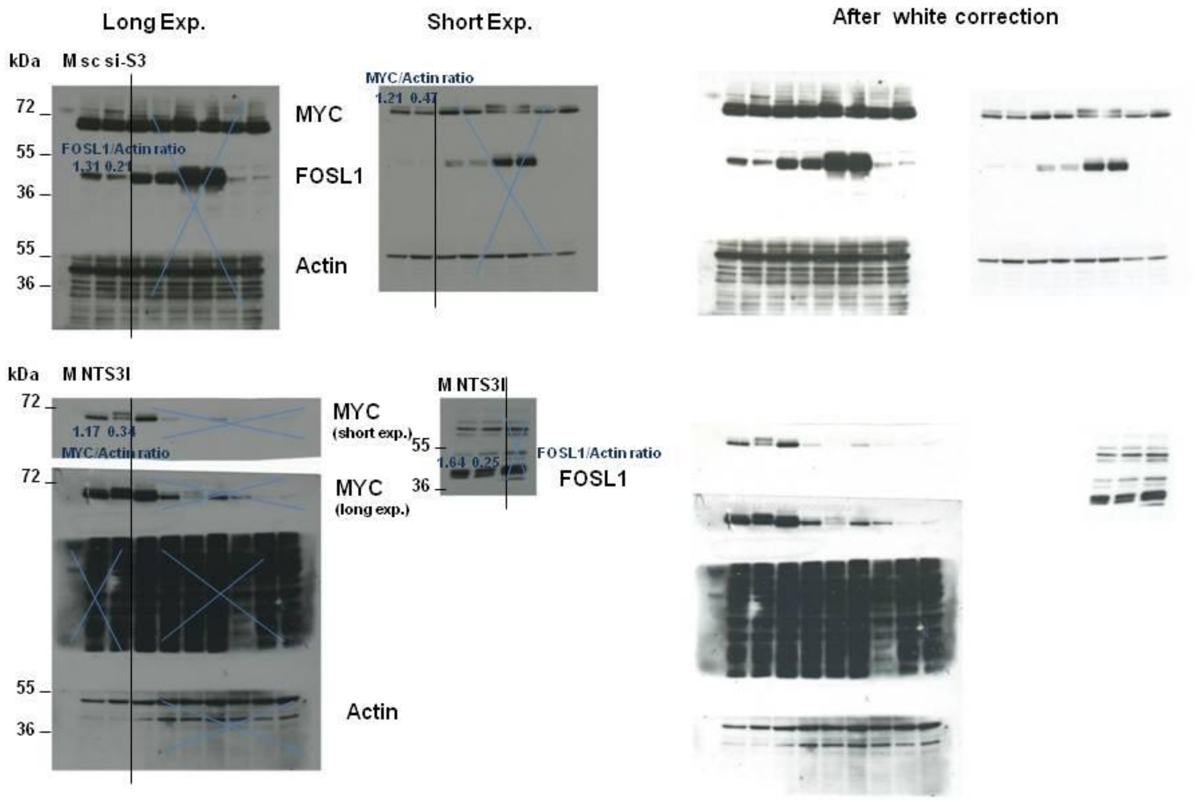

Supplementary Figure S19. Whole western-blots corresponding to Supplementary Figure S5 (A), S7 (B), S11 (C), S15 (D) and densitomentric quantification of each bands relative to actin control bands. Crossed-out samples should not be considered.
