# Supplementary material for: Hyper-Activation of STAT3 Sustains Progression of Non-Papillary Basal-Type Bladder Cancer via FOSL1 Regulome
Source: Cancers (Basel). 2019 Aug 21;11(9):1219. doi: 10.3390/cancers11091219 (PMC6770563; doi:10.3390/cancers11091219)
Supplement: Supplementary file 1 [file cancers-11-01219-s001.zip › cancers-565379-supplementary/Benerini Gatta et al. Supplementary File 2.pdf]

Supplementary File 2

Hyper-activation of STAT3 sustains progression of non-papillary basal-type bladder cancer via FOSL1 regulome

Luisa Benerini Gatta [1,,2,3,†], Laura Melocchi [4,†], Mattia Bugatti [1,3], Francesco Missale [5], Silvia Lonardi [1,3], Benedetta Zanetti [1,3], Luca Cristinelli [2,3], Sandra Belotti [2,3], Claudio Simeone [2,3], Roberto Ronca [1], Elisabetta Grillo [1], Sara Licini [1,3], Debora Bresciani [1,3], Regina Tardanico [1,3], Szeman Ruby Chan [6], Emanuele Giurisato [7,8], Stefano Calza [1,†] and William Vermi [1,3,9,*]

Supplementary Tables

Supplementary Table S1 reports the details of the clinical and pathological features of UBC patients and correlation with pSTAT3 expression.

Supplementary Table S2 reports the details of the patients characteristics of MIBCs (retrospective UBC cohort).

Supplementary Table S3 reports the details of the immunohistochemistry scores of basal and luminal markers on MIBCs (retrospective UBC cohort).

Supplementary Table S4 reports the details of the patients characteristics and pSTAT3 score of NMIBCs (retrospective UBC cohort).

Supplementary Table S5 reports the details of the TaqMan™ Gene Expression Assays.

Supplementary Table S6 illustrates the data from gene expression profile of STAT3-regulated transcripts in human UBC cell lines.

Supplementary Table S7 reports the details of the coefficients for classification using elastic-net (STAT3 signature, TCGA dataset).

Supplementary Table S8 reports the details of the Hazard Ratios (HR) for univariate Cox models testing overall survival (STAT3 signature, TCGA dataset).

Supplementary Table S9 reports the details of the Fold Change (FC) for Luminal *vs* Basal comparison in FOSL1 signature (TCGA dataset and GSE32894 dataset).

Supplementary Table S10 reports the details of the Fold Change (FC) for MIBCs *vs* NMIBCs comparison in FOSL1 signature (GSE32894 dataset).

Supplementary Table S11 reports the details of the coefficients for MIBCs *vs* NMIBCs classification using FOSL1 signature (GSE32894 dataset).

Supplementary Table S12 reports the details of antibodies used for immunohistochemistry and western blotting.

Supplementary Table S1. Clinical and pathological features of UBC patients and correlation with pSTAT3 expression

Patient characteristics			pSTAT3 Low	pSTAT3 High	
		n. (%)	n. (%)	n. (%)	p value
**All**		193 (100)	119 (62)	74 (38)	
**Gender**					0,173
	**Male**	159 (82)	102 (64)	57 (36)	
	**Female**	34 (18)	17 (50)	17 (50)	
**Age**					0,4582
	**≥ Median**	104 (54)	67 (64)	37 (36)	
	**≤ Median**	89 (46)	52 (58)	37 (42)	
**Histology (TURB)**					0,0019*
	**LGPBC**	25 (13)	22 (88)	3 (12)	
	**NMIBC**	79 (41)	52 (66)	27 (34)	
	**MIBC**	89 (46)	45 (51)	44 (49)	
**pT stage**					0,0007*
	**pTa-Tis**	53 (27)	43 (36)	10 (14)	
	**pT1**	51 (26)	31 (26)	20 (27)	
	**pT2**	28 (15)	10 (8)	18 (24)	
	**pT3-T4**	61 (32)	35 (29)	26 (35)	
**N stage**					1,0
	**N0**	164 (85)	101 (62)	63 (38)	
	**N+**	29 (15)	18 (62)	11 (38)	
**AJCC stage**					0,0014*
	**Stage 0**	53 (27)	43 (36)	10 (14)	
	**Stage I**	51 (26)	31 (26)	20 (27)	
	**Stage II**	24 (12)	9 (8)	15 (20)	
	**Stage III-IV**	65 (34)	36 (30)	29 (39)	
**Grading (NMIBC)**					0,0036*
	**High Grade**	79 (76)	97 (58)	71 (42)	
	**Low Grade**	25 (24)	22 (88)	3 (12)	
**Early stage**					0,0184*
	**Ta-Tis**	53 (51)	44 (81)	10 (19)	
	**T1**	51 (49)	30 (60)	20 (40)	
**Subtype (MIBC)**					< 0,0001
	**Luminal**	42 (47)	31 (74)	11 (26)	
	**Basal**	22 (25)	3 (14)	19 (86)	
	**Non Type**	25 (28)	11 (44)	14 (56)	
**CIS concomitant**					0,3787
	**No**	94 (49)	61 (65)	33 (35)	
	**Yes**	99 (51)	58 (59)	41 (41)	

TURB: Trans-urethral resection biopsy

pT stage: pathological staging on following cystectomy if performed

LGPBC: Low grade papillary urothelial bladder cancer

NMIBC: Non-muscle invasive bladder cancer

MIBC: Muscle invasive bladder cancer

CIS: Carcinoma in situ.

Supplementary Table S2. Patients characteristics of MIBCs (retrospective UBC cohort).

Case	G	Age	D	pTN	AJCC	CP	Grade	CIS	Divergent Differentiation
**#1**	M	51	N	pT2a N0	II	N	HG	N	SCC, sarcomatoid
**#2**	M	68	Y	pT2b N0	II	N	HG	N	SCC, sarcomatoid
**#3**	M	82	Y	pT4 N0	III	N	HG	N	Sarcomatoid
**#4**	M	73	N	pT3b N0	III	Y	HG	N	SCC
**#5**	M	68	N	pT3b N1	IV	N	HG	Y	N
**#6**	M	57	N	pT2b N0	II	N	HG	Y	SCC
**#7**	M	52	N	pT2b N0	II	N	HG	N	Glandular
**#8**	M	78	N	pT3a N0	III	N	HG	Y	Sarcomatoid
**#9**	M	73	N	pT3a N0	III	N	HG	Y	Sarcomatoid, glandular
**#10**	F	54	N	pT2b N0	II	N	HG	Y	N
**#11**	M	78	N	pT3a N0	III	N	HG	Y	SCC
**#12**	F	70	N	pT2b N0	II	N	HG	Y	Micropapillary
**#13**	M	51	N	pT2b N0	II	N	HG	Y	SCC, sarcomatoid
**#14**	M	61	N	pT2 N0	II	N	HG	Y	SCC
**#15**	M	69	N	pT2b N0	II	N	HG	N	SCC, micropapillary
**#16**	M	71	N	pT3 N0	III	N	HG	N	SCC, micropapillary, [illegible]
**#17**	M	76	N	pT3a N0	III	N	HG	Y	Micropapillary
**#18**	M	56	N	pT3a N0	III	N	HG	N	Nested
**#19**	M	65	N	pT4a N0	III	N	HG	Y	N
**#20**	M	69	Y	pT4 N0	III	Y	HG	N	SCC
**#21**	M	73	Y	pT3b N2	IV	Y	HG	Y	Sarcomatoid, glandular, NE
**#22**	F	90	Y	pT2b N0	II	N	HG	N	N
**#23**	M	57	Y	pT3a N0	III	Y	HG	N	Glandular
**#24**	M	75	N	pT3a N1	IV	Y	HG	Y	N
**#25**	F	80	ND	pT2b N1	IV	Y	HG	Y	N
**#26**	M	60	Y	pT3a N0	III	Y	HG	Y	N
**#27**	M	65	Y	pT2b N0	II	N	HG	Y	N
**#28**	M	76	Y	pT3a N1	IV	Y	HG	Y	N
**#29**	M	65	N	pT2a N0	II	Y	HG	Y	Nested
**#30**	M	57	N	pT3a N0	III	Y	HG	N	NE, SCC, sarcomatoid, [illegible]
**#31**	F	86	Y	pT3b N0	III	Y	HG	N	Nested, SCC, sarcomatoid
**#32**	M	62	N	pT4 N0	III	Y	HG	Y	Micropapillary, glandular
**#33**	M	70	N	pT2b N0	II	Y	HG	Y	N
**#34**	M	68	Y	pT3a N2	IV	Y	HG	N	SCC, micropapillary
**#35**	M	59	N	pT2a N0	II	Y	HG	N	N
**#36**	M	66	Y	pT3a N0	III	Y	HG	N	N
**#37**	M	76	Y	pT2b N0	II	Y	HG	Y	N
**#38**	M	61	Y	pT2b N0	II	Y	HG	Y	Sarcomatoid
**#39**	M	75	Y	pT3b N2	IV	Y	HG	Y	Micropapillary
**#40**	F	81	Y	pT3a N3	IV	Y	HG	Y	N
**#41**	M	74	Y	PT3a N0	III	Y	HG	N	N
**#42**	M	64	Y	pT4b N0	IV	N	HG	Y	Micropapillary, signet ring
**#43**	M	73	Y	pT4b N0	IV	N	HG	Y	Rabdoid
**#44**	F	82	Y	pT4 N1	IV	Y	HG	Y	N
**#45**	M	74	N	pT3a N0	III	Y	HG	Y	N
**#46**	F	70	Y	pT3b N2	IV	N	HG	N	SCC
**#47**	F	61	Y	pT3b N1	IV	Y	HG	Y	Micropapillary, pagetoid

**#48**	M	67	N	pT2 N0	II	N	HG	Y	N
**#49**	M	77	N	pT2a N0	II	Y	HG	N	Nested
**#50**	M	84	N	pT3a N0	III	N	HG	Y	SCC
**#51**	M	65	N	pT2b N1	IV	M	HG	N	N
**#52**	M	81	Y	pT4 N0	III	Y	HG	Y	N
**#53**	M	84	Y	pT3a N2	IV	Y	HG	Y	Trophoblastic
**#54**	M	76	Y	pT3a N1	IV	Y	HG	N	N
**#55**	M	82	N	pT3a N0	III	Y	HG	Y	Nested
**#56**	F	80	Y	pT3a N1	IV	Y	HG	N	N
**#57**	M	85	Y	pT3a N0	III	Y	HG	Y	N
**#58**	M	65	N	pT2b N0	II	N	HG	N	N
**#59**	M	72	N	pT3a N0	III	Y	HG	Y	Glandular
**#60**	F	47	Y	pT4 N0	III	Y	HG	N	N
**#61**	M	85	N	pT4 N1	IV	N	HG	Y	SCC, basaloid
**#62**	M	81	N	pT4 N0	III	N	HG	Y	N
**#63**	M	77	N	pT2a N0	II	N	HG	Y	N
**#64**	M	83	N	pT2a N0	II	N	HG	Y	N
**#65**	M	87	N	pT2b N0	II	N	HG	Y	SCC
**#66**	M	74	N	pT3a N0	III	N	HG	N	Sarcomatoid
**#67**	M	72	N	pT2a N1	IV	N	HG	Y	Micropapillary, pagetoid
**#68**	M	72	N	pT2 N0	II	N	HG	Y	N
**#69**	M	69	Y	pT3a N2	IV	N	HG	Y	Micropapillary
**#70**	M	79	Y	pT3a N1	IV	N	HG	N	N
**#71**	M	72	N	pT2 N0	II	N	HG	Y	N
**#72**	F	41	N	pT4a N1	IV	Y	HG	N	SCC
**#73**	M	85	Y	pT4a N0	III	N	HG	N	Micropapillary, sarcomatoid
**#74**	M	80	Y	pT3a N1	IV	N	HG	N	N
**#75**	F	82	Y	pT3a N1	IV	N	HG	N	NE, limphoepithelioma-like
**#76**	M	54	N	pT3a N0	III	Y	HG	Y	Sinciziale
**#77**	M	66	N	pT3a N2	IV	Y	HG	Y	N
**#78**	M	82	N	pT4 N1	IV	N	HG	Y	N
**#79**	F	79	Y	pT3a N0	III	Y	HG	N	N
**#80**	M	49	N	pT4 N0	III	Y	HG	N	N
**#81**	F	82	Y	pT3a N0	III	N	HG	Y	N
**#82**	M	79	N	pT3a N0	III	N	HG	Y	Micropapillary
**#83**	M	65	N	pT4 N2	IV	N	HG	N	Plasmocytoid, signet ring
**#84**	M	65	Y	pT4a N2	IV	Y	HG	Y	N
**#85**	M	79	N	pT3a N0	III	N	HG	N	Micropapillary
**#86**	M	71	Y	pT4 N0	III	N	HG	Y	N
**#87**	M	85	Y	pT2 N1	IV	N	HG	Y	N
**#88**	M	73	Y	pT3a N1	IV	Y	HG	Y	SCC
**#89**	F	75	N	pT3a N2	IV	Y	HG	Y	N

pT: T staging on TURB (trans-urethral resection biopsy)

CP: clinical progression (Y: yes, N: no, ND: not determined)

pTN: Staging on following cystectomy

HG: high grade; LG: low grade

CIS: Concomitant presence of carcinoma in situ (yes/no)

Supplementary Table S3. MIBC subtype by immunohistochemistry score (retrospective UBC cohort).

Case	Age	G	pSTAT3	CK5	CK14	CK20	UPK2	MYC	FOSL1	Subtype	pTN	Stag
#1	51	M	2	0	0	1	2	-	-	Luminal	pT2a N0	II
#2	68	M	3	3	0	0	0	3	3	Basal	pT2b N0	II
#3	82	M	3	3	1	0	0	2	3	Basal	pT4 N0	III
#4	73	M	3	3	2	0	0	3	2	Basal	pT3b N0	III
#5	68	M	1	0	0	1	1	-	-	Luminal	pT3b N1	IV
#6	57	M	2	2	0	0	2	-	-	Heterog	pT2b N0	II
#7	52	M	1	3	0	1	0	1	3	Basal	pT2b N0	II
#8	78	M	3	1	1	1	1	-	-	Heterog	pT3a N0	III
#9	73	M	3	3	1	0	0	3	2	Basal	pT3a N0	III
#10	54	F	2	0	0	2	3	2	1	Luminal	pT2b N0	II
#11	78	M	2	2	0	1	1	-	-	Heterog	pT3a N0	III
#12	70	F	2	0	0	3	3	2	1	Luminal	pT2b N0	II
#13	51	M	3	2	3	0	0	2	3	Basal	pT2b N0	II
#14	61	M	2	3	3	1	0	3	3	Basal	pT2 N0	II
#15	69	M	1	1	0	3	3	3	2	Luminal	pT2b N0	II
#16	71	M	1	2	0	1	2	-	-	Heterog	pT3 N0	III
#17	76	M	0	3	0	0	0	0	0	Basal	pT3a N0	III
#18	56	M	0	3	0	2	1	-	-	Heterog	pT3a N0	III
#19	65	M	1	0	1	2	2	1	1	Luminal	pT4a N0	III
#20	69	M	3	3	2	0	0	3	3	Basal	pT4 N0	III
#21	73	M	1	0	0	1	0	-	-	Negativ	pT3b N2	IV
#22	90	F	3	3	2	0	0	2	3	Basal	pT2b N0	II
#23	57	M	2	2	0	3	2	-	-	Heterog	pT3a N0	III
#24	75	M	1	0	0	0	3	1	0	Luminal	pT3a N1	IV
#25	80	F	2	0	0	2	2	3	0	Luminal	pT2b N1	IV
#26	60	M	2	0	0	0	0	-	-	Negativ	pT3a N0	III
#27	65	M	1	0	0	1	2	-	-	Luminal	pT2b N0	II
#28	76	M	1	0	1	3	3	0	1	Luminal	pT3a N1	IV
#29	65	M	1	1	0	3	3	-	-	Luminal	pT2a N0	II
#30	57	M	3	2	2	1	0	2	3	Basal	pT3a N0	III
#31	86	F	3	3	0	1	0	1	0	Basal	pT3b N0	III
#32	62	M	1	0	0	3	2	0	0	Luminal	pT4 N0	III
#33	70	M	1	0	0	2	2	-	-	Luminal	pT2b N0	II
#34	68	M	2	2	1	2	2	-	-	Heterog	pT3a N2	IV
#35	59	M	1	1	0	3	3	1	1	Luminal	pT2a N0	II
#36	66	M	3	3	2	0	1	2	3	Basal	pT3a N0	III
#37	76	M	2	2	1	2	1	-	-	Heterog	pT2b N0	II
#38	61	M	3	1	1	2	1	-	-	Heterog	pT2b N0	II
#39	75	M	1	0	0	1	2	-	-	Luminal	pT3b N2	IV
#40	81	F	1	1	0	1	3	0	0	Luminal	pT3a N3	IV
#41	74	M	3	3	0	2	0	-	-	Heterog	PT3a N0	III
#42	64	M	1	1	0	1	2	-	-	Luminal	pT4b N0	IV
#43	73	M	0	0	2	3	3	-	-	Heterog	pT4b N0	IV
#44	82	F	1	1	0	3	3	-	-	Luminal	pT4 N1	IV
#45	74	M	0	0	0	3	3	2	2	Luminal	pT3a N0	III
#46	70	F	2	3	0	2	0	-	-	Heterog	pT3b N2	IV
#47	61	F	1	0	0	3	3	0	0	Luminal	pT3b N1	IV

**#48**	67	M	2	1	0	0	0	-	-	Negativ	pT2 N0	II
**#49**	77	M	1	0	0	3	3	1	1	Luminal	pT2a N0	II
**#50**	84	M	1	3	1	1	2	-	-	Heterog	pT3a N0	III
**#51**	65	M	2	3	1	1	0	3	3	Basal	pT2b N1	IV
**#52**	81	M	3	2	1	1	2	-	-	Heterog	pT4 N0	III
**#53**	84	M	2	3	2	0	0	2	3	Basal	pT3a N2	IV
**#54**	76	M	1	2	1	2	1	-	-	Heterog	pT3a N1	IV
**#55**	82	M	1	0	1	0	3	-	-	Luminal	pT3a N0	III
**#56**	80	F	1	1	3	1	3	-	-	Heterog	pT3a N1	IV
**#57**	85	M	1	1	0	3	2	-	-	Luminal	pT3a N0	III
**#58**	65	M	2	1	0	3	1	-	-	Luminal	pT2b N0	II
**#59**	72	M	2	0	0	1	2	-	-	Luminal	pT3a N0	III
**#60**	47	F	3	3	2	0	0	3	3	Basal	pT4 N0	III
**#61**	85	M	2	3	1	0	0	3	1	Basal	pT4 N1	IV
**#62**	81	M	1	3	3	2	2	-	-	Heterog	pT4 N0	III
**#63**	77	M	2	1	0	3	3	-	-	Luminal	pT2a N0	II
**#64**	83	M	1	1	0	2	3	-	-	Luminal	pT2a N0	II
**#65**	87	M	2	2	1	1	2	-	-	Heterog	pT2b N0	II
**#66**	74	M	3	3	2	0	0	2	3	Basal	pT3a N0	III
**#67**	72	M	3	1	0	3	3	0	1	Luminal	pT2a N1	IV
**#68**	72	M	2	2	1	2	3	-	-	Heterog	pT2 N0	II
**#69**	69	M	2	1	0	3	3	0	2	Luminal	pT3a N2	IV
**#70**	79	M	1	1	0	0	1	-	-	Heterog	pT3a N1	IV
**#71**	72	M	0	0	0	3	3	1	1	Luminal	pT2 N0	II
**#72**	41	F	3	3	3	0	0	3	2	Basal	pT4a N1	IV
**#73**	85	M	1	3	3	0	0	2	3	Basal	pT4a N0	III
**#74**	80	M	1	0	0	3	3	1	0	Luminal	pT3a N1	IV
**#75**	82	F	0	0	0	0	0	-	-	Negativ	pT3a N1	IV
**#76**	54	M	0	0	0	3	3	1	1	Luminal	pT3a N0	III
**#77**	66	M	0	0	0	3	3	2	2	Luminal	pT3a N2	IV
**#78**	82	M	1	2	0	0	3	-	-	Heterog	pT4 N1	IV
**#79**	79	F	3	3	2	0	0	3	3	Basal	pT3a N0	III
**#80**	49	M	1	0	1	3	3	-	-	Luminal	pT4 N0	III
**#81**	82	F	2	0	0	3	3	1	0	Luminal	pT3a N0	III
**#82**	79	M	1	0	0	3	2	-	-	Luminal	pT3a N0	III
**#83**	65	M	1	0	0	2	3	-	-	Luminal	pT4 N2	IV
**#84**	65	M	2	0	0	3	3	1	1	Luminal	pT4a N2	IV
**#85**	79	M	1	0	0	1	3	-	-	Luminal	pT3a N0	III
**#86**	71	M	0	0	0	1	1	-	-	Luminal	pT4 N0	III
**#87**	85	M	1	0	0	3	3	-	-	Luminal	pT2 N1	IV
**#88**	73	M	2	3	1	0	0	1	2	Basal	pT3a N1	IV
**#89**	75	F	1	0	0	3	3	-	-	Luminal	pT3a N2	IV

Heterog = “non-type” UBC

Supplementary Table S4. Patients characteristics and pSTAT3 score of NMIBCs (retrospective UBC cohort).

Case	G	A	pT	pTN	AJCC Stage	CP	Grade	CIS	pSTAT3
#1	M	48	pTa	NA	0	N	LG	N	0
#2	M	66	pTa	NA	0	N	LG	N	3
#3	F	37	pTa	NA	0	N	LG	N	3
#4	M	75	pTa	NA	0	N	LG	N	1
#5	M	58	pTa	NA	0	N	LG	N	2
#6	F	65	pTa	NA	0	N	LG	N	1
#7	M	78	pTa	NA	0	N	LG	N	0
#8	F	58	pTa	NA	0	N	LG	N	0
#9	M	48	pTa	NA	0	N	LG	N	1
#10	M	70	pTa	NA	0	N	LG	N	0
#11	M	62	pTa	NA	0	N	LG	N	1
#12	M	45	pTa	NA	0	N	LG	N	0
#13	M	72	pTa	NA	0	N	LG	N	0
#14	M	80	pTa	NA	0	N	LG	N	0
#15	M	70	pTa	NA	0	N	LG	N	0
#16	F	59	pTa	NA	0	N	LG	N	0
#17	M	81	pTa	NA	0	N	LG	N	1
#18	M	73	pTa	NA	0	N	LG	N	0
#19	M	62	pTa	NA	0	N	LG	N	0
#20	M	51	pTa	NA	0	N	LG	N	0
#21	M	83	pTa	NA	0	N	LG	N	0
#22	M	30	pTa	NA	0	N	LG	N	0
#23	F	76	pTa	NA	0	N	LG	N	1
#24	M	71	pTa	NA	0	N	LG	N	0
#25	M	55	pTa	NA	0	N	LG	N	1
#26	M	75	pT1	pT2b	I	Y	HG	N	1
#27	M	75	pT1	pT3a	I	Y	HG	Y	0
#28	M	69	pTis	pT4	0	Y	HG	Y	0
#29	M	69	pT1	pT2b	I	Y	HG	N	2
#30	F	72	pTa	pT3b	0	Y	HG	N	1
#31	M	64	pT1	pT2b	I	Y	HG	Y	1
#32	F	74	pT1	pT3b	I	Y	HG	N	1
#33	M	60	pT1	pT2b	I	Y	HG	Y	1
#34	F	69	pTa	pT3a	0	Y	HG	N	3
#35	M	69	pT1	pT3a	I	Y	HG	N	0
#36	F	62	pT1	pT3a	I	Y	HG	Y	2
#37	F	80	pT1	pT3b	I	Y	HG	Y	1
#38	M	78	pTa	pT3b	0	Y	HG	Y	2
#39	M	80	pT1	pT2a	I	Y	HG	N	1
#40	M	53	pT1	pT4b	I	Y	HG	Y	2
#41	M	84	pT1	pT4	I	Y	HG	Y	1
#42	F	87	pTa	pT2	0	Y	HG	N	1
#43	M	72	pT1	pT4	I	Y	HG	Y	1
#44	M	56	pT1	pT4	I	Y	HG	Y	2
#45	M	69	pT1	pT3a	I	Y	HG	Y	1
#46	M	87	pT1	pT4	I	Y	HG	Y	2

**#47**	M	75	pTa	pT4a N0	0	Y	HG	N	2
**#48**	M	75	pT1	pT2b N0	I	Y	HG	Y	0
**#49**	F	66	pT1	pT4a N[illegible]	I	Y	HG	Y	3
**#50**	M	66	pT1	pT3a N1	I	Y	HG	N	1
**#51**	M	62	pTa	pT3a N0	0	Y	HG	N	0
**#52**	M	66	pT1	pT1	I	Y	HG	N	1
**#53**	F	67	pT1	pT1	I	Y	HG	N	1
**#54**	M	67	pT1	pTis	I	N	HG	Y	1
**#55**	M	74	pT1	pTis	I	Y	HG	Y	2
**#56**	F	81	pTa	pT1	0	N	HG	N	0
**#57**	F	72	pT1	pTa	I	N	HG	Y	0
**#58**	M	78	pT1	pT1	I	N	HG	N	1
**#59**	M	58	pT1	pT1	I	N	HG	Y	1
**#60**	M	87	pT1	pT1	I	Y	HG	Y	1
**#61**	M	75	pTa	pT1	0	Y	HG	Y	1
**#62**	M	67	pT1	pT1	I	Y	HG	Y	2
**#63**	M	69	pT1	pT1	I	Y	HG	Y	2
**#64**	M	64	pTis	pT1	0	Y	HG	Y	0
**#65**	M	76	pTa	pT1	0	N	HG	N	2
**#66**	M	74	pT1	pT1	I	N	HG	Y	1
**#67**	M	70	pTa	pTis	0	N	HG	Y	2
**#68**	M	52	pTa	pTa	0	N	HG	N	1
**#69**	F	74	pT1	pTis	I	N	HG	Y	3
**#70**	F	68	pT1	pTis	I	N	HG	Y	2
**#71**	M	69	pT1	pT1	I	Y	HG	Y	1
**#72**	M	55	pT1	pT1	I	N	HG	N	3
**#73**	M	76	pTa	pTis	I	N	HG	Y	1
**#74**	M	64	pTis	pTis	0	N	HG	Y	1
**#75**	M	68	pT1	pT1	I	N	HG	Y	2
**#76**	M	66	pT1	pTis	I	N	HG	Y	1
**#77**	M	63	pTa	NA	0	N	HG	N	0
**#78**	M	62	pT1	NA	I	N	HG	Y	0
**#79**	M	82	pT1	NA	I	N	HG	N	0
**#80**	M	74	pTa	NA	0	N	HG	N	0
**#81**	M	70	pTa	NA	0	N	HG	N	1
**#82**	M	80	pTa	NA	0	N	HG	N	3
**#83**	M	80	pT1	NA	I	N	HG	N	2
**#84**	M	68	pTa	NA	0	N	HG	N	1
**#85**	M	78	pT1	NA	I	N	HG	N	1
**#86**	M	57	pT1	NA	I	N	HG	N	0
**#87**	M	81	pT1	NA	I	N	HG	N	3
**#88**	M	69	pTa	NA	0	N	HG	N	2
**#89**	M	86	pT1	NA	I	N	HG	N	3
**#90**	M	67	pT1	NA	I	N	HG	Y	0
**#91**	M	52	pT1	NA	I	N	HG	N	1
**#92**	M	72	pT1	NA	I	N	HG	N	0
**#93**	M	75	pTa/Tis	NA	0	N	HG	Y	0
**#94**	M	72	pT1	NA	I	N	HG	N	2
**#95**	M	51	pTis	NA	0	N	HG	Y	0

**#96**	M	78	pT1	NA	I	N	HG	Y	2
**#97**	M	75	pTis	NA	0	N	HG	Y	1
**#98**	M	70	pTa	NA	0	N	HG	N	1
**#99**	M	53	pTis	NA	0	N	HG	Y	0
**#100**	M	68	pT1	NA	I	N	HG	Y	0
**#101**	F	56	pT1	NA	I	N	HG	Y	2
**#102**	M	69	pT1	NA	I	N	HG	Y	3
**#103**	M	65	pTis	NA	0	N	HG	Y	0
**#104**	M	76	pTa	NA	0	N	HG	N	1

pT: T staging on TURB (trans-urethral resection biopsy)

CP: clinical progression (Y: yes, N: no)

pTN: Staging on following cystectomy if performed

HG: high grade; LG: low grade

NA: not applicable

CIS: Concomitant presence of carcinoma in situ (yes/no)

Supplementary Table S5. TaqMan™ Gene Expression Assays.

Gene	Cat. no.
IL6	Hs00174132_m1
IL1B	Hs01555410_m1
SOCS1	Hs00864158_g1
SOCS3	Hs01000485_g1
STAT3	Hs00374280_m1
MYC	Hs00153408_m1
FOS	Hs00170630_m1
FOSL1	Hs04187685_m1
TP53	Hs01034249_m1
NFKB1	Hs00765730_m1
TWIST	Hs00361186_m1
SNAI1	Hs00195591_m1
NANOG	Hs02387400_g1
POU5F1	Hs00999632_g1
HIF1A	Hs00153153_m1
BCL2	Hs00608023_m1
BCL2L1	Hs00236329_m1
MCL1	Hs01050896_m1
BNIP3	Hs00969291_m1
BIRC5	Hs00153353_m1
CCDN1	Hs00765553_m1
CD44	Hs01075861_m1
KRT14	Hs03044364_m1
MMP1	Hs00899658_m1
MMP9	Hs00957562_m1
VEGFA	Hs00900055_m1
HPRT1	4333768T

TaqMan™ Gene Expression Assays were from Applied Biosystems, Thermo Fisher Scientific.

Supplementary Table S6. Fold Change analysis of the expression of STAT3-regulated transcripts in UBC cell lines.

Gene	*RT4_vs_5637_STAT3*		*si-STAT3_vs_si-scrambled 5637*		*si-STAT3_vs_si-scrambled RT4*		*si-STAT3_vs_si-scrambled HT-1376*		*si-STAT3_vs_si-scrambled T24*	
	FC	FDR	FC	FDR	FC	FDR	FC	FDR	FC	FDR
IL6	0,02752	0,00097	1,30586	0,29872	1,81504	0,05553	0,97265	0,91888	1,24401	0,46184
IL1B	0,1169	1,8E-05	0,84675	0,40529	1,15269	0,74606	1,54756	0,10239	1,6529	0,11474
SOCS1	1,07425	0,62903	1,65864	0,21276	1,81504	0,13627	1,07923	0,83819	1,8025	0,20298
SOCS3	0,5535	0,10817	0,69496	0,25002	0,94278	0,94597	1,68179	0,1463	1,08298	0,78396
STAT3	0,51883	0,00365	0,1155	0,0017	0,16724	0,00606	0,23245	0,02195	0,12763	0,0047
MYC	0,2885	0,0002	0,79692	0,25446	1,12054	0,76453	0,98623	0,92092	1,05702	0,78396
FOS	9,9406	1E-05	0,57435	0,02046	1,02456	0,94597	0,5946	0,0393	1,47939	0,11905
FOSL1	0,08617	1,8E-05	0,60082	0,05413	1,00696	0,96601	1,09429	0,83819	0,87661	0,56348
TP53	0,24599	1,8E-05	1,47939	0,18425	1,02101	0,94597	1,53156	0,15352	1,31494	0,34684
NFKB1	1,10957	0,04967	1,20999	0,20493	1,04608	0,86792	1,61328	0,02195	1,01045	0,91422
TWIST1	7,4E-05	5E-07	1,02101	0,89865	0,59874	0,05553	1,7593	0,0393	1,37078	0,27433
SNAI1	0,21714	0,00052	1,46409	0,36536	1,81504	0,24895	1,10957	0,83819	1,19334	0,73418
NANOG	1,56917	0,00371	1,40444	0,76355	1,54756	0,86792	1,34723	0,83819	0,38024	0,46184
POU5F1	1,87905	0,00173	1,6529	0,01786	1,06807	0,85672	1,11729	0,67323	1,12117	0,47616
HIF1A	6,40856	0,00063	1,75564	0,18425	0,10844	0,00061	1,56374	0,31257	1,47939	0,34684
BCL2	1,53688	0,00475	0,62851	0,27874	0,75786	0,74606	0,87661	0,83819	0,59255	0,30959
BCL2L1	0,64767	0,00329	1,21841	0,29872	0,72951	0,13627	1,08298	0,83819	1,16878	0,46184
MCL1	0,51169	0,00066	0,46652	0,00102	0,8827	0,4908	1,12506	0,50344	1,34723	0,11474
BNIP3	0,33294	0,00329	0,68777	0,58267	0,92019	0,94597	0,42928	0,35855	0,9298	0,91422
BIRC5	0,74226	0,03655	1,10573	0,55609	1,07923	0,85672	1,6077	0,0393	1,10957	0,56348
CCND1	1,00463	0,95685	1,03886	0,8135	1,66324	0,03562	1,3566	0,15752	1,42899	0,12914
CD44	0,20781	2,6E-05	0,87055	0,24696	1,04972	0,85672	0,96929	0,83819	1,24401	0,11905
KRT14	0,02616	6,5E-05	0,39338	0,02029	0,39915	0,0296	0,92338	0,83819	1,48968	0,30959
MMP1	0,00121	1,9E-06	0,40613	0,30453	1,08673	0,94597	0,56841	0,75387	0,78458	0,78396
MMP9	0,00367	8,9E-06	1,14076	0,60303	1,81504	0,06446	1,09051	0,83819	0,80107	0,46184
VEGFA	0,88782	0,62903	0,69834	0,30453	1,07549	0,94597	0,81225	0,75387	1,38992	0,46184

Supplementary Table S7: coefficients for UBC subtype (Basal, Luminal) classification using elastic-net for STAT3 targets (TCGA cohort). Scaled coefficients report the relative importance (magnitude) of coefficients on absolute scale. *Genes selected via stability path variable selection.

	**Coefficients (scaled)**	**Coefficients (value)**
**STAT3***	100.00	-1.13
**BIRC5***	62.33	-0.71
**CD44***	51.13	-0.58
**SOCS1***	37.99	-0.43
**MYC***	26.88	-0.30
**POU5F1**	16.55	0.19
**MMP9**	11.77	-0.13
**KRT14***	11.19	-0.13
**HIF1A**	8.61	-0.099
**SNAI1**	7.75	-0.088
**FOS**	7.68	0.087
**SOCS3**	5.25	-0.060
**FOSL1**	4.19	-0.048

Supplementary Table S8: Hazard Ratios (HR) for univariate Cox models testing overall survival in STAT3 signature TCGA dataset.

	**HR**	**p-value**
BCL2	1.011	0.881
BCL2L1	0.992	0.944
BIRC5	1.029	0.704
BNIP3	0.992	0.885
CCND1	0.986	0.745
CD44	1.084	0.090
FOS	1.127	0.020
FOSL1	1.132	0.008
HIF1A	1.161	0.054
IL1B	0.995	0.898
IL6	1.043	0.133
KRT14	1.038	0.030
MCL1	1.164	0.158
MMP1	0.988	0.666
MMP9	1.069	0.030
MYC	1.139	0.010
NANOG	0.931	0.144
NFKB1	0.931	0.545
POU5F1	0.891	0.006
SNAI1	1.119	0.036
SOCS1	0.997	0.955
SOCS3	1.136	0.028
STAT3	1.327	0.024
TP53	1.055	0.488
TWIST1	1.108	0.007
VEGFA	0.880	0.050

Supplementary Table S9. Fold Change (FC) for Luminal *vs* Basal comparison in FOSL1 signature in TCGA and GSE32894 datasets. .

	TCGA			GSE32894		
	**FC**	**P-value**	**FDR**	**FC**	**P-value**	**FDR**
PLAUR	0.252	< 1e-04	< 1e-04	0.426	< 0.001	< 0.001
IL1RAP	0.247	< 1e-04	< 1e-04	0.449	< 0.001	< 0.001
HMGA2	0.030	< 1e-04	< 1e-04	0.919	< 0.001	< 0.001
PLEKHA6	4.032	< 1e-04	< 1e-04	2.338	< 0.001	< 0.001
LAMA3	0.088	< 1e-04	< 1e-04	0.427	< 0.001	< 0.001
CLCF1	0.304	< 1e-04	< 1e-04	0.623	< 0.001	< 0.001
GDF15	7.160	< 1e-04	< 1e-04	2.367	< 0.001	< 0.001
FOSL1	0.259	< 1e-04	< 1e-04	0.662	< 0.001	< 0.001
IL7R	0.194	< 1e-04	< 1e-04	0.375	< 0.001	< 0.001
SRGN	0.255	< 1e-04	< 1e-04	0.276	< 0.001	< 0.001
SNAI2	0.260	< 1e-04	< 1e-04	0.411	< 0.001	< 0.001
MMP9	0.138	< 1e-04	< 1e-04	0.273	< 0.001	< 0.001
AXL	0.299	< 1e-04	< 1e-04	0.517	< 0.001	< 0.001
CCNA2	0.468	< 1e-04	< 1e-04	0.544	< 0.001	< 0.001
INHBA	0.189	< 1e-04	< 1e-04	-	-	-
IL22RA2	0.162	< 1e-04	< 1e-04	0.908	< 0.001	< 0.001
PTP4A1	0.487	< 1e-04	< 1e-04	0.728	< 0.001	< 0.001
ADORA2B	0.305	< 1e-04	< 1e-04	0.509	< 0.001	< 0.001
IFI30	0.390	< 1e-04	< 1e-04	0.421	< 0.001	< 0.001
VEGFC	0.355	< 1e-04	< 1e-04	0.574	< 0.001	< 0.001
PLAU	0.283	< 1e-04	< 1e-04	0.271	< 0.001	< 0.001
CCL5	0.232	< 1e-04	< 1e-04	0.413	< 0.001	< 0.001
TGFB1	0.439	< 1e-04	< 1e-04	-	-	-
PPP2R3A	0.487	< 1e-04	< 1e-04	0.920	0.11072	0.14306
BIRC5	0.488	< 1e-04	< 1e-04	0.793	< 0.001	< 0.001
FOXM1	0.455	< 1e-04	< 1e-04	0.725	< 0.001	< 0.001
HBEGF	0.416	< 1e-04	< 1e-04	0.422	< 0.001	< 0.001
CCL20	0.172	< 1e-04	< 1e-04	0.302	< 0.001	< 0.001
IL6	0.182	< 1e-04	< 1e-04	0.435	< 0.001	< 0.001
BIRC3	0.342	< 1e-04	< 1e-04	0.773	< 0.001	< 0.001
COL7A1	0.321	< 1e-04	< 1e-04	0.338	< 0.001	< 0.001
ITGB5	0.527	< 1e-04	< 1e-04	0.674	< 0.001	< 0.001
MMP1	0.191	< 1e-04	< 1e-04	0.212	< 0.001	< 0.001
CXCL8	0.246	< 1e-04	< 1e-04	0.088	< 0.001	< 0.001
IGFBP3	3.067	< 1e-04	< 1e-04	1.565	< 0.001	< 0.001
FEN1	0.595	< 1e-04	< 1e-04	0.839	0.00803	0.01247
SERPINE2	0.297	< 1e-04	< 1e-04	0.375	< 0.001	< 0.001
MX1	0.398	< 1e-04	< 1e-04	0.679	0.00187	0.00325

AURKB	0.543	< 1e-04	< 1e-04	0.625	< 0.001	< 0.001
SERPINE1	0.323	< 1e-04	< 1e-04	0.540	< 0.001	< 0.001
SFN	0.382	< 1e-04	< 1e-04	0.409	< 0.001	< 0.001
PHLDA1	0.439	< 1e-04	< 1e-04	0.339	< 0.001	< 0.001
APLN	0.391	< 1e-04	< 1e-04	0.824	< 0.001	< 0.001
VDR	0.533	< 1e-04	< 1e-04	0.862	0.00137	0.00242
ZEB2	0.497	< 1e-04	< 1e-04	0.699	< 0.001	< 0.001
RGS5	2.193	< 1e-04	< 1e-04	1.529	< 0.001	< 0.001
MCM10	0.545	< 1e-04	< 1e-04	0.616	< 0.001	< 0.001
LAMB3	0.310	< 1e-04	< 1e-04	0.906	0.00951	0.01439
MT1F	0.465	< 1e-04	< 1e-04	0.659	< 0.001	< 0.001
MTDH	0.689	< 1e-04	< 1e-04	0.824	< 0.001	< 0.001
BGLAP	1.787	< 1e-04	< 1e-04	1.041	0.15248	0.18654
ABHD11	1.598	< 1e-04	< 1e-04	1.119	0.03694	0.05446
THBD	0.416	< 1e-04	< 1e-04	0.768	< 0.001	< 0.001
VEGFD	2.406	< 1e-04	< 1e-04	-	-	-
MCM2	0.625	< 1e-04	< 1e-04	0.837	0.08606	0.11644
COL1A1	0.375	< 1e-04	< 1e-04	0.340	< 0.001	< 0.001
PAICS	0.738	< 1e-04	< 1e-04	0.980	0.70974	0.76281
SEC14L1	0.768	< 1e-04	< 1e-04	0.943	0.04355	0.06260
CCL2	0.464	< 1e-04	< 1e-04	0.365	< 0.001	< 0.001
HHIP	3.005	< 1e-04	< 1e-04	1.063	0.07007	0.09709
CLCA2	0.174	< 1e-04	< 1e-04	0.176	< 0.001	< 0.001
VSIR	0.635	< 1e-04	< 1e-04	-	-	-
COL1A2	0.392	< 1e-04	< 1e-04	0.417	< 0.001	< 0.001
KRT18	1.793	< 1e-04	< 1e-04	0.951	0.12535	0.15841
CHML	0.694	< 1e-04	< 1e-04	0.989	0.65197	0.72092
GFRA1	2.842	< 1e-04	< 1e-04	1.055	0.03950	0.05749
DMTF1	1.274	< 1e-04	< 1e-04	1.085	0.08786	0.11748
NFATC1	0.571	< 1e-04	< 1e-04	0.946	0.19991	0.23701
LIF	0.521	< 1e-04	< 1e-04	0.796	< 0.001	< 0.001
ADGRG6	1.832	< 1e-04	< 1e-04	1.528	< 0.001	< 0.001
H2AFZ	0.768	< 1e-04	< 1e-04	0.830	0.00227	0.00384
C3	0.428	< 1e-04	< 1e-04	0.893	< 0.001	< 0.001
VCAN	0.469	< 1e-04	< 1e-04	0.366	< 0.001	< 0.001
JUN	0.646	< 1e-04	< 1e-04	0.696	0.00128	0.00231
EZH2	0.739	< 1e-04	< 1e-04	0.805	< 0.001	0.00133
EP300	1.284	< 1e-04	< 1e-04	1.103	0.02838	0.04238
JUND	1.341	< 1e-04	< 1e-04	1.162	0.00262	0.00428
IVL	2.954	< 1e-04	< 1e-04	1.031	0.34274	0.39078
PADI2	0.567	< 1e-04	< 1e-04	0.996	0.86177	0.90921
VEGFB	0.796	< 1e-04	< 1e-04	0.891	0.00548	0.00875
MED20	0.863	< 1e-04	0.000135	1.002	0.97028	0.97028
KCNIP1	0.601	0.000129	0.000196	0.999	0.93819	0.94642

MMP10	0.392	0.000158	0.000236	0.350	< 0.001	< 0.001
TJAP1	1.141	0.000192	0.000283	1.147	0.00261	0.00428
NPNT	0.626	0.000208	0.000303	0.850	< 0.001	0.00136
NTRK2	0.500	0.000219	0.000316	0.958	0.06729	0.09437
DCN	0.560	0.000232	0.000331	0.646	< 0.001	< 0.001
ITGB4	0.699	0.000354	0.000499	0.909	0.25892	0.30383
E2F1	0.739	0.000384	0.000535	-	-	-
VEGFA	1.354	0.000457	0.000629	1.029	0.67201	0.73601
SP1	1.136	0.000503	0.000685	1.201	< 0.001	< 0.001
NFE2L3	0.744	0.001155	0.001556	1.013	0.88684	0.91880
USF2	1.114	0.001799	0.002399	1.035	0.14486	0.18108
MMP2	0.665	0.001911	0.002521	0.850	< 0.001	< 0.001
ZEB1	0.760	0.002921	0.003812	0.941	0.00714	0.01125
CHMP1A	0.899	0.004091	0.005284	1.008	0.87273	0.91240
TIMP3	0.688	0.004305	0.005504	0.964	0.71737	0.76387
PLCD4	0.862	0.007974	0.010090	0.972	0.10491	0.13710
CENPM	0.847	0.015799	0.019788	0.744	< 0.001	< 0.001
HMOX1	0.768	0.016384	0.020316	0.705	0.00208	0.00357
DLC1	1.234	0.020100	0.024677	1.635	< 0.001	< 0.001
MEF2C	0.831	0.026392	0.032085	0.965	0.44929	0.50655
NPR3	0.757	0.031772	0.038250	0.968	0.17693	0.21417
YTHDF1	1.060	0.049325	0.058811	1.081	0.05853	0.08309
MIR222	0.867	0.055176	0.065161	-	-	-
TXLNG	0.927	0.076845	0.089894	1.060	0.10228	0.13520
NFATC2	0.853	0.080720	0.093545	-	-	-
OLFM1	0.827	0.140167	0.160933	0.747	< 0.001	< 0.001
NFATC3	0.932	0.145071	0.165035	1.002	0.92944	0.94611
NOS3	0.906	0.189405	0.213511	0.816	< 0.001	< 0.001
RGS17	1.155	0.212951	0.237891	1.133	0.34321	0.39078
CHAF1A	0.941	0.217252	0.240529	1.185	< 0.001	< 0.001
CCND1	0.864	0.257271	0.282315	0.801	0.12298	0.15714
GJA1	0.884	0.279507	0.304025	0.670	0.00264	0.00428
ABI3BP	0.872	0.311302	0.335664	0.953	0.47398	0.52920
SMTN	0.945	0.421139	0.450183	0.918	0.08525	0.11644
PTGES	0.922	0.513231	0.543937	0.734	0.00941	0.01439
HPD	0.939	0.555979	0.584249	-	-	-
RRP1	1.025	0.617314	0.643251	0.991	0.68015	0.73790
MGP	0.932	0.635638	0.656826	0.865	0.30721	0.35686
JUNB	0.985	0.820709	0.841057	1.139	0.15143	0.18654
MIR221	0.978	0.860449	0.874555	-	-	-
SCD	1.001	0.989317	0.995624	1.164	0.19036	0.22803
SH3GL1	1.000	0.995624	0.995624	0.996	0.92966	0.94611

Supplementary Table S10. Fold Change (FC) for MIBCs *vs* NMIBCs comparison in FOSL1 signature in GSE32894 dataset.

	FC	P Value	FDR
COL1A1	2.5356047	< 1e-05	< 1e-05
PLAUR	1.6939748	< 1e-05	< 1e-05
MCM10	1.9861748	< 1e-05	< 1e-05
FOXM1	1.5092869	< 1e-05	< 1e-05
BIRC5	1.3451223	< 1e-05	< 1e-05
VCAN	2.3451953	< 1e-05	< 1e-05
IFI30	1.8159113	< 1e-05	< 1e-05
AURKB	1.9607332	< 1e-05	< 1e-05
CXCL8	4.0862641	< 1e-05	< 1e-05
COL1A2	2.1468790	< 1e-05	< 1e-05
CCNA2	1.8150385	< 1e-05	< 1e-05
MMP9	2.3378038	< 1e-05	< 1e-05
PLAU	2.1297195	< 1e-05	< 1e-05
CCL2	2.0923924	< 1e-05	< 1e-05
PLEKHA6	0.6022658	< 1e-05	< 1e-05
FEN1	1.4193298	< 1e-05	< 1e-05
EZH2	1.4019611	< 1e-05	< 1e-05
IL7R	1.8368629	< 1e-05	< 1e-05
MCM2	1.7070360	< 1e-05	< 1e-05
SRGN	2.0954416	< 1e-05	< 1e-05
IL6	1.6705135	< 1e-05	< 1e-05
CLCF1	1.3731463	< 1e-05	< 1e-05
SERPINE2	2.1707132	< 1e-05	< 1e-05
CENPM	1.3860942	< 1e-05	< 1e-05
ZEB2	1.2901079	< 1e-05	< 1e-05
MMP2	1.1522453	< 1e-05	< 1e-05
H2AFZ	1.2997705	< 1e-05	< 1e-05
AXL	1.4417805	< 1e-05	< 1e-05
CCL20	1.8682514	< 1e-05	< 1e-05
HMOX1	1.5851166	< 1e-05	< 1e-05
VEGFC	1.4023960	< 1e-05	< 1e-05
NOS3	1.2668029	< 1e-05	1.03003e-05

BIRC3	1.1657742	< 1e-05	1.12629e-05
SP1	0.8496119	< 1e-05	1.43182e-05
JUNB	0.7039388	< 1e-05	1.90995e-05
MTDH	1.2087539	2.43526e-05	7.77931e-05
EP300	0.8511734	2.92649e-05	9.09584e-05
PHLDA1	1.5731298	4.88431e-05	0.000147815
CCL5	1.5771552	8.64296e-05	0.000254857
COL7A1	1.6366861	0.000111652	0.000320998
APLN	1.1630767	0.000174709	0.000490037
DCN	1.3423417	0.000201906	0.000541316
SERPINE1	1.4557155	0.000202405	0.000541316
IL1RAP	1.2799186	0.000209241	0.000546881
SFN	1.3939012	0.000333871	0.000853227
ADGRG6	0.7317863	0.000422183	0.001055457
VEGFB	1.1358593	0.000447666	0.001095352
LIF	1.1335650	0.000515445	0.001234921
DLC1	0.7195023	0.000631467	0.001482014
OLFM1	1.1860706	0.001061441	0.002441314
MMP1	1.7035717	0.001545547	0.003485058
MGP	1.4570076	0.002104273	0.004653680
ZEB1	1.0622405	0.002159986	0.004686761
LAMA3	1.2958991	0.003231305	0.006881483
ITGB5	1.2119039	0.003721702	0.007781740
PLCD4	1.0419211	0.007619738	0.015446957
HBEGF	1.2511455	0.007656318	0.015446957
ITGB4	0.8228962	0.008124898	0.016109711
RGS5	0.7959596	0.010092188	0.019671214
PPP2R3A	1.1222053	0.010638770	0.020390977
FOSL1	1.1607263	0.016596782	0.031289015
CCND1	0.7445787	0.018699606	0.034684754
HMGA2	1.0450083	0.020268533	0.036998116
IL22RA2	1.0516271	0.021156131	0.038014924
RGS17	0.7759614	0.026582717	0.047030961
NPNT	1.0955297	0.030236721	0.052685195
SEC14L1	1.0558761	0.031675213	0.054367903

MT1F	1.2474916	0.034142063	0.057346711
LAMB3	0.9322459	0.034408027	0.057346711
IVL	0.9433530	0.038944063	0.063979532
PAICS	1.1000697	0.040711108	0.065940527
ABHD11	0.9122425	0.049692950	0.079370685
SMTN	1.0876454	0.051381904	0.080944095
CHAF1A	1.0842479	0.055132418	0.085678758
THBD	1.1137228	0.059717184	0.091566349
SNAI2	1.1813526	0.070997513	0.107430447
KCNIP1	1.0257257	0.076926195	0.114889771
NFATC1	1.0662703	0.087890583	0.129582270
SH3GL1	1.0646409	0.112642786	0.163973676
IGFBP3	0.8438425	0.120629007	0.173404197
MEF2C	1.0618542	0.141317307	0.200635683
CHML	1.0312656	0.146083385	0.204873040
TIMP3	0.8806859	0.151968053	0.209677631
NFATC3	1.0355408	0.153155835	0.209677631
GDF15	0.8085161	0.160209303	0.211647922
PTP4A1	1.0794655	0.160737491	0.211647922
MMP10	1.2397214	0.161431277	0.211647922
TXLNG	0.9573697	0.161956670	0.211647922
PADI2	0.9745484	0.184868247	0.238874702
KRT18	0.9646337	0.208595116	0.266538204
GJA1	1.1486759	0.236562325	0.298952388
PTGES	1.1292170	0.246181436	0.307726795
BGLAP	0.9723532	0.250170630	0.309350779
CHMP1A	1.0491821	0.255239851	0.312261520
NFE2L3	1.0935953	0.270272637	0.323863520
USF2	0.9774063	0.270355634	0.323863520
SCD	1.1096228	0.300996748	0.356851815
HHIP	0.9724626	0.340046766	0.399034470
JUN	1.0950054	0.361011260	0.419356514
DMTF1	1.0370924	0.382099813	0.439414785
MX1	1.0929092	0.416318492	0.474026005
ADORA2B	1.0739785	0.435365075	0.490852780

GFRA1	0.9830053	0.451584723	0.504196535
C3	1.0170307	0.472762539	0.519935773
JUND	0.9690746	0.474723967	0.519935773
ABI3BP	1.0409124	0.485382936	0.526594695
TJAP1	0.9727566	0.491806027	0.528576571
VEGFA	0.9632603	0.515371387	0.548775088
VDR	1.0227873	0.578790279	0.608510930
MED20	1.0201096	0.582053933	0.608510930
CLCA2	1.0942259	0.689413382	0.714257108
YTHDF1	1.0057187	0.874628424	0.898055971
NTRK2	1.0020352	0.921898242	0.938215025
NPR3	1.0014968	0.942467891	0.944931823
RRP1	0.9986337	0.944931823	0.944931823

Supplementary Table S11. Coefficients for MIBCs *vs* NMIBCs classification using FOSL1 signature in GSE32894 dataset. Scaled coefficients reports the relative importance (magnitude) of coefficients on absolute scale *Genes with more stable selection path.

	**Coefficients (scaled)**	**Coefficients (value)**
KCNIP1*	100.00	0.53
CHML	54.30	0.29
NTRK2*	53.39	-0.28
BIRC5*	52.20	0.28
MMP2	46.59	0.25
MTDH*	37.38	0.120
DMTF1*	34.47	0.18
PLCD4	31.53	0.17
EP300	30.11	-0.17
EZH2	29.30	0.15
VEGFB	26.85	0.14
NOS3	26.68	0.14
PPP2R3A	24.87	0.13
FOXM1	24.10	0.13
PLAUR*	23.60	0.12
VCAN*	21.92	0.12
CLCF1	21.25	0.11
FEN1	18.58	0.10
CHMP1A	18.39	0.10
AURKB	17.31	0.09
MCM10	16.95	0.09
COL1A1	16.92	0.09
COL1A2	16.60	0.09
PADI2	16.56	-0.09
SP1	16.14	-0.09
MCM2	12.38	0.07
CCNA2	11.92	0.06
MED20	11.90	0.06
IL1RAP	10.03	0.05
H2AFZ	9.83	0.05
RGS5	9.76	-0.05
JUNB	9.76	-0.05
MX1*	9.58	-0.05
PLAU	8.99	0.05
SERPINE1	8.70	0.05
ABHD11	8.62	-0.05
IFI30	8.41	0.04
PLEKHA6	8.10	-0.04

ITGB4	7.88	-0.04
CXCL8	7.84	0.04
VEGFC	7.07	0.04
CCL20	6.43	0.03
DLC1	6.35	-0.03
SRGN	5.69	0.03
MMP9	5.60	0.030
NFATC3	5.24	0.028
VDR	4.80	0.025
CCND1	4.09	-0.022
IL7R	4.01	0.021
HMOX1	3.42	0.018
SERPINE2	3.12	0.016
ADGRG6	2.71	-0.014
PAICS	2.55	0.013
CCL2	2.32	0.012
CENPM	2.05	0.011
ZEB2	1.82	0.0096
LIF	1.72	0.0090
COL7A1	1.27	0.0067
MMP10	1.22	-0.0064
PTGES	0.90	0.0047
GDF15	0.60	-0.0032
TIMP3	0.51	-0.0027
MGP	0.06	0.0003

Supplementary Table S12. IHC and western blotting antibodies.

Antibody	Clone	Isotype	Company	Dilution
Actin	polyclonal	Rabbit	Sigma-Aldrich	1:1000
CD3	LN10	Mouse IgG1	Leica Biosystems	1:100
CD44	DF1485	Mouse IgG2a	Thermo Fisher Scientific	1:200
CD66b	G10F5	Mouse IgM	Biolegend	1:200
CD163	10D6	Mouse IgG1	Thermo Fisher Scientific	1:50
CK 5/6	D5/16B4	Mouse IgG1	Thermo Fisher Scientific	1:100
CK 14	LL002	Mouse IgG3	Leica Biosystems	1:50
CK 20	Ks20.8	Mouse IgG2a	Leica Biosystems	1:50
FOSL1 (FRA-1)	C-12	Mouse IgG1	Santa Cruz	1:1000 WB-1:80 IHC
MYC	Y69	Rabbit	Abcam	1:10000 WB- 1:75 IHC
pSTAT3 (Tyr705)	D3A7	Rabbit IgG	Cell Signaling	1:1000 WB- 1:120 IHC
Sma	1A4(asm-	mouse IgG2a	Thermo Fisher Scientific	1:100
STAT3	84	Mouse IgG	BD Biosciences	1:2500
UPK2	BC21	Mouse IgG1	Biocare Medical	1:50
Isotype control	DA1E	Rabbit IgG	Cell Signaling	1:2500
Isotype control	11711	Mouse IgG1	R&D	1:400

WB= western blotting; IHC= immunohistochemistry
